# Gastropods alien to South Africa cause severe environmental harm in their global alien ranges across habitats

**DOI:** 10.1002/ece3.4385

**Published:** 2018-07-22

**Authors:** David Kesner, Sabrina Kumschick

**Affiliations:** ^1^ Department of Botany & Zoology Centre for Invasion Biology Stellenbosch University Matieland South Africa; ^2^ Invasive Species Programme South African National Biodiversity Institute Kirstenbosch National Botanical Gardens Claremont South Africa

**Keywords:** environmental impact, environmental impact classification for alien taxa, Gastropoda, impact assessment, resilience, risk analysis, socioeconomic impact, socio‐economic impact classification for alien taxa

## Abstract

Alien gastropods have caused extensive harm to biodiversity and socioeconomic systems like agriculture and horticulture worldwide. For conservation and management purposes, information on impacts needs to be easily interpretable and comparable, and the factors that determine impacts understood. This study aimed to assess gastropods alien to South Africa to compare impact severity between species and understand how they vary between habitats and mechanisms. Furthermore, we explore the relationship between environmental and socioeconomic impacts, and both impact measures with life‐history traits. We used the Environmental Impact Classification for Alien Taxa (EICAT) and Socio‐Economic Impact Classification for Alien Taxa (SEICAT) to assess impacts of 34 gastropods alien to South Africa including evidence of impact from their entire alien range. We tested for correlations between environmental and socioeconomic impacts per species, and with fecundity and native latitude range using Kendall's tau tests. Kruskal–Wallis tests were used to compare impact magnitude among mechanisms and habitats, respectively. This study presents the first application of EICAT and SEICAT for invertebrates. There was no correlation between environmental impacts and socioeconomic impacts. Habitats did not differ regarding the severity of impacts recorded, but impacts via disease transmission were lower than other mechanisms. Neither fecundity nor native range latitude was correlated with impact magnitude. Despite gastropods being agricultural and horticultural pests globally, resilience of socioeconomic systems makes high impacts uncommon. Environmental systems may be vulnerable to gastropod impacts across habitats, having experienced multiple local extinctions of wetland island snail fauna. South Africa stands out as the only continental country that follows this trend. The knowledge gained on severity and nature of gastropod impacts is useful in risk assessment, which can aid conservation management. To make impact assessments more realistic, we suggest alternative ways of reporting impacts classified under EICAT and SEICAT.

## 1 INTRODUCTION

Alien species introductions continue to be a major driver of global change (Banks, Paini, Bayliss, & Hodda, 2015; Hulme, 2015). Alien impacts on native systems are diverse in magnitude and nature, causing alterations to environments and socioeconomic networks (Jeschke et al., 2014). The complexity of alien impacts makes them difficult to understand, let alone manage (Keller & Kumschick, 2017; Ricciardi, Hoopes, Marchetti, & Lockwood, 2013; Simberloff et al., 2013). Recent targets aimed at controlling harmful aliens and preventing their spread and impacts have been imposed globally, such as the Aichi Target 9 that aims to control or eradicate priority species by 2020 (CBD, 2013). Such targets will be challenging to achieve without a better understanding of alien impact across taxa and habitats.

Gastropods are a taxonomic class that represents significant problem as aliens, causing a multitude of environmental and socioeconomic impacts in many habitats. In Hawai'i, they have been implicated as a major threat to native plants (Joe & Daehler, 2008) and snails (Curry et al., 2016; Meyer & Cowie, 2010), and have been suggested to harm native species in aquatic habitats (Darwall, Smith, Tweddle, & Skelton, 2009; Miranda & Perissinotto, 2012). Further, many gastropods have been the cause of significant problems in agriculture (Barker, 2002; Coupland & Baker, 1995; Nash & Hoffmann, 2012), resulting in economic losses by reducing yield and leading to rejection of exports (Charwat, Davies, & Fraser, 1995; Cowie, Hayes, Tran, & Meyer, 2008; Moran, Gotlib, & Yaakov, 2004). Moreover, they have been implicated as playing a role in the transmission of diseases to humans (Senanayake, Pryor, Walker, & Konecny, 2003).

Given the complex nature of alien impacts, the need for universal metrics to quantify impacts on environmental and socioeconomic systems is evident. These have been provided by various impact scoring schemes, including the Environmental Impact Classification for Alien Taxa (EICAT; Blackburn et al., 2014; Hawkins et al., 2015) and more recently the Socio‐Economic Impact Classification for Alien Taxa (SEICAT; Bacher et al., 2017). These schemes provide standardized methods to compare impacts across taxa and habitats (Evans, Kumschick, & Blackburn, 2016). A convenient property of these schemes is that their impact classification frameworks are similar to the IUCN Red List (Kumschick et al., 2017), and EICAT has recently been adopted by the IUCN as a framework for impact scoring of alien taxa (https://portals.iucn.org/congress/motion/014). This allows for alien impact metrics to be seamlessly integrated into existing management and policy procedures (Bacher et al., 2017). Environmental and socioeconomic impacts have been shown to be similar in severity for a wide array of taxa (mammals: Kumschick, Bacher, & Blackburn, 2013; arthropods, plants, and fish: Kumschick et al., 2015), but there are exceptions (amphibians: Bacher et al., 2017; birds: Kumschick et al., 2013).

Impacts are highly context‐dependent, and they can differ between recipient habitats and over time (e.g., Kumschick et al., 2014). Impact scoring schemes can aid in understanding generalizability and context dependency of impacts, for example, by comparing impact magnitudes across habitats and impact mechanisms. This has been done for various taxa, with some mechanisms showing to be more important than others. For example, alien birds, amphibians, and fish cause more severe impacts via predation compared with other mechanisms (Evans et al., 2016; Kumschick et al., 2015; Measey et al., 2016). An investigation of impact severity by habitat type is yet to be undertaken.

To increase not only our basic understanding of patterns related to how impact magnitude differs between taxa and regions, it has been suggested that life‐history traits could aid moving toward a more predictive understanding of impacts, notwithstanding novel introductions (Kumschick et al., 2014). Such analyses have been undertaken for a wide array of taxa (Evans et al., 2016; Kumschick et al., 2013; Nentwig, Kühnel, & Bacher, 2010; Novoa, Kumschick, Richardson, Rouget, & Wilson, 2016). According to the impact equation given by Parker et al. (1999), abundance is proportional to severity of impact of invaders, under the rationale that any biomass controlled by an invader represents resources no longer available to natives. Therefore, the ability of an alien to reproduce rapidly and attain higher abundances should correlate with their impacts. This has been supported by significant correlations between impact and fecundity for some taxa, namely mammals and mollusks (Keller, Drake, & Lodge, 2007; Nentwig et al., 2010). High fecundity has been cited as a characteristic of many harmful gastropods (Charwat et al., 1995; Nash & Hoffmann, 2012; Rumi, Sánchez, & Ferrando, 2010), and mollusks with higher fecundity have been shown to have higher probabilities of causing environmental and economic damage in the US great lakes (Keller et al., 2007).

Ecological flexibility is also expected to play an important role with regard to impact because species that can live under diverse environmental circumstances may sustain higher population densities and occupy larger areas (Nentwig et al., 2010). The size of a native range should be proportional to the diversity of habitats in which a species can persist and therefore increase the likely similarity between native and alien ranges (Kumschick et al., 2013). Moreover, the likelihood of a species being transported, dispersed, and causing impact in novel ranges is higher if a species is more widely distributed (Moodley, Geerts, Richardson, & Wilson, 2013; Novoa et al., 2016). The latitude range of a species’ native range has been used as a measure of ecological flexibility and has shown to correlate with impact for birds and mammals (Kumschick et al., 2013) as well as cactaceae (Novoa et al., 2016), suggesting that this may be an important trait that correlates with alien impact magnitude.

Given the wide variety of alien gastropod impacts globally, it is of relevance to assess these within impact classification schemes, as well as identify potential patterns with respect to the severity of their impacts. This can aid the prioritization of management in countries and regions where information on impact is limited, as in South Africa. More generally, this knowledge may be integrated into risk analysis tools, ultimately aiding in the identification of effective management actions (Keller & Kumschick, 2017). Here, we present the first global assessment of environmental and socioeconomic impacts of alien gastropods, by applying the EICAT and SEICAT scoring schemes to the gastropods alien to South Africa. Because South Africa has experienced introductions of globally problematic alien gastropods across aquatic and terrestrial habitats (Barker, 2002; Griffiths & Picker, 2011; Herbert, 2010; Kappes, Delgado, Alonso, & Ibáñez, 2009; Perera & Valderrama, 2010), it provides a representative case study upon which to base a global analysis of alien gastropod impact. We test whether environmental impact severities of alien gastropods are correlated with their socioeconomic impacts, and whether there are differences in impact severity among habitats and impact mechanisms, respectively. Further, we assess whether fecundity and native range size are correlated with gastropod impacts. Lastly, we are interested in whether there is a potential publication bias toward more studies being performed on species achieving higher maximum impact scores, which could influence the overall species classifications as suggested in the guidelines for using the two schemes.

## 2 METHODS

A list of 34 gastropods alien to South Africa was compiled from Griffiths and Picker (2011) and Herbert (2010) (Table 1). The native range of each species was identified in accordance with published literature, including the following sources: Terrestrial Mollusc Tool (http://idtools.org/id/mollusc/index.php); Invasive Species Compendium (https://www.cabi.org/isc/); Encyclopaedia of Life (http://eol.org/); and the Carnegie Museum of Natural History website (http://www.carnegiemnh.org/science/mollusks/index.html; for the list of native ranges as well as the literature used, see Supporting Information Table S1 and Appendix S1).

**Table 1:** Results of the EICAT and SEICAT assessments for gastropods in order of decreasing EICAT maximum impact

Species	EICAT maximum impact	Mechanism(s)	EICAT confidence	EICAT no. publications	SEICAT maximum impact	Constituent(s) of human well‐being	SEICAT confidence	SEICAT no. publications
*Helisoma duryi*	MR	Competition	Low	12	DD			
*Oxychilus draparnaudi*	MR	Predation	Low	6	DD			
*Tarebia granifera*	MR	Competition	Low	36	MN	Material and immaterial goods for good life	Low	2
*Theba pisana*	MR	Competition	Low	7	MO	Material and immaterial goods for good life	Low	24
*Deroceras laeve*	MO	Grazing/herbivory/browsing	Low	8	MN	Health; Material and immaterial goods for good life; Social, spiritual and cultural relations	High	9
*Deroceras panormitanum*	MO	Grazing/herbivory/browsing; Predation	Low	5	MN	Material and immaterial goods for good life	High	10
*Lehmannia nyctelia*	MO	Grazing/herbivory/browsing	Low	3	DD			
*Limacus flavus*	MO	Grazing/herbivory/browsing	Low	3	MN	Material and immaterial goods for good life	Medium	5
*Limax maximus*	MO	Grazing/herbivory/browsing; Competition	Low	7	MN	Health; Material and immaterial goods for good life; Social, spiritual and cultural relations	Low	9
*Milax gagates*	MO	Grazing/herbivory/browsing; Competition	Low	6	MN	Material and immaterial goods for good life	High	12
*Oxychilus alliarius*	MO	Predation	Low	9	MN	Health	Low	1
*Oxychilus cellarius*	MO	Predation	Low	4	DD			
*Aplexa marmorata*	MN	Competition	Low	1	MN	Health	Low	1
*Arion intermedius*	MN	Grazing/herbivory/browsing; Competition	Low	3	MN	Material and immaterial goods for good life	Medium	5
*Bradybaena similaris*	MN	Transmission of diseases to native species	Low	3	MN	Health; Material and immaterial goods for good life	High	18
*Cochlicella barbara*	MN	Grazing/herbivory/browsing	Low	5	MO	Material and immaterial goods for good life	Medium	12
*Cornu aspersum*	MN	Transmission of diseases to native species	Low	3	MN	Health; Material and immaterial goods for good life; Social, spiritual and cultural relations	High	11
*Deroceras reticulatum*	MN	Grazing/herbivory/browsing; Transmission of diseases to native species; Predation	Low	9	MN	Health; Material and immaterial goods for good life; Social, spiritual and cultural relations	High	35
*Lehmannia valentiana*	MN	Transmission of diseases to native species; Predation	Medium	3	MN	Health; Material and immaterial goods for good life	Medium	7
*Littorina saxatilis*	MN	Predation	Low	1	DD			
*Lymnaea columella*	MN	Competition	Low	2	MN	Material and immaterial goods for good life	Low	9
*Physa acuta*	MN	Transmission of diseases to native species; Competition	Low	3	MN	Health; Material and immaterial goods for good life	Low	3
*Radix rubiginosa*	MN	Transmission of diseases to native species	Low	2	MC	Social, spiritual and cultural relations	Low	1
*Vallonia costata*	MN	Transmission of diseases to native species	Low	1	DD			
*Vallonia pulchella*	MC	Transmission of diseases to native species	Low	3	MC	Material and immaterial goods for good life	Low	2
*Zonitoides arboreus*	MC	Transmission of diseases to native species	Low	2	MN	Health; Material and immaterial goods for good life; Social, spiritual and cultural relations	High	11
*Arion hortensis*	DD				MN	Material and immaterial goods for good life	High	15
*Eobania vermiculata*	DD				MO	Material and immaterial goods for good life	Medium	7
*Gyraulus chinensis*	DD				DD			
*Discus rotundatus*	DD				DD			
*Thais blanfordi*	DD				DD			
*Lauria cylindracea*	DD				DD			
*Cochlicopa cf. lubricella*	DD				DD			
*Cochlicopa cf. lubrica*	DD				DD			

*Note*

Impacts for EICAT are described as follows: MC—discernible impacts, but not deleterious to individuals; MN—fitness of individuals is reduced; MO—declines in population sizes of at least one species; MR—local extinctions of at least one species; MV—irreversible changes to community composition or extinctions; DD—data deficient. Impacts for SEICAT are described as follows: MC—discernible impacts, but not deleterious to individual persons; MN—well‐being of individual people is reduced; MO—change to activity sizes; MR—local disappearance of an activity; MV—irreversible disappearance of an activity; DD—data deficient. Impact classifications and mechanisms/constituents of human well‐being refer to the maximum impact reported. Confidence scores and number of publications related to all the information available on a species’ impacts not only the mechanism/constituent with maximum impact. Detailed information on the literature used to assign scores is available in Supporting Information Appendix S1 and S2.

A literature search including publications up to August 2017 was conducted for each species, using Google Scholar and Scopus, with the scientific binomial species name as the search term. A filter was applied to Scopus, which included the following fields: “Agricultural and Biological Sciences,” “Medicine, Immunology and Microbiology,” “Environmental Science,” “Veterinary, Pharmacology, Toxicology and Pharmaceutics” and “Multidisciplinary.” Titles and abstracts were screened regarding their relevance for gastropod impacts, and the search considered complete when the literature begun to repeat itself, no further information on impact was found (usually within the first 100 search results), or the search engine ran out of results.

### 2.1 Impact classification schemes

EICAT has eight categories into which an alien can be assigned (Hawkins et al., 2015). Each category is defined by verbal descriptions that make EICAT robust toward assessor bias, and universally applicable across taxa. Five categories describe successive impact scenarios involving increasing scales of native biological organization affected and hence increasing impact magnitude. These categories (in order of increasing impact) are as follows: Minimal Concern (MC), Minor (MN), Moderate (MO), Major (MR), Massive (MV) (Hawkins et al., 2015; for descriptions of each category, see Table 1). The remaining three categories, Data Deficient (DD), No Alien Population (NA) and Not Evaluated (NE), do not reflect the impact status of an alien, but describe cases where there is a lack of adequate information on impact or no need to quantify it. An alien may receive multiple EICAT scores, depending on the amount and depth of literature found on its impact. Each EICAT score is assigned a mechanism, describing the nature of impact. EICAT has twelve such mechanisms, these being competition, predation, hybridization, transmission of diseases to native species, parasitism, poisoning/toxicity, biofouling, grazing/herbivory/browsing, chemical, physical or structural impact on ecosystem, and interaction with other alien species (Hawkins et al., 2015).

SEICAT is similar to EICAT, having an identical impact classification framework. However, instead of using the scale of biological organization impacted as a means of quantifying environmental impact, it uses the scale of human activity impacted as a common metric to evaluate impact on human well‐being (Bacher et al., 2017). For example, a Minor impact (MN) is described as individual people experiencing difficulties in taking part in an activity, which can be detected through income loss or health problems. A Massive impact (MV) is the local disappearance of an activity from the area that an alien invades (termed a “regime shift”). As with EICAT, SEICAT classifies each alien by its maximum potential impact. Analogous to EICAT mechanisms, SEICAT has four main categories termed “constituents of human well‐being”: health; safety; material and immaterial goods for good life; and social, spiritual, and cultural relations (Bacher et al., 2017).

Scoring under both schemes is based on the best available evidence, and scores can change if more information on an alien taxon becomes available (Blackburn et al., 2014). EICAT and SEICAT make use of an identical confidence rating system giving an indication of the probability of the assessment being correct and assigning each score a confidence level of low, medium or high based on the reliability of the source, scale of the study and other factors (Bacher et al., 2017; Hawkins et al., 2015).

We assigned environmental and socioeconomic impacts identified from the literature to the EICAT and SEICAT classification schemes respectively (lists of the literature sources used are given in Supporting Information Appendix S2 and S3). Each impact record was assigned a habitat based on where the impact occurred as per the Hawkins et al. (2015) EICAT habitat classification scheme. Impacts identified under synonymous species names were included. Studies from the global alien range were included, but studies describing impacts in the native range of a species were not scored under EICAT, except for cryptogenic species. Regardless of a species’ alien/native status, all socioeconomic impacts identified were scored under SEICAT. This is because artificial habitats are becoming increasingly homogenized globally (McKinney, 2006), making impacts in the native artificial systems adequate surrogates for potential socioeconomic impacts in invaded ranges. This was confirmed by a nonpaired Wilcoxon test showing no difference between all records found for socioeconomic impact scores in the native (*N* = 117) and alien range (*N* = 59; *W* = 3402, *p *=* *0.82). Only primary references, except for one case where the direct quotation was provided and the primary reference was inaccessible, were included.

For species that were assigned impact scores, further literature and database searches were conducted for data on their fecundity. This was done using the same databases that were used for native range information. When searching the literature, the scientific binomial species name followed by “fecundity” was used as the search term. We further consulted gastropod experts for additional literature on fecundity (for fecundity data and the literature used, see Supporting Information Table S1 and Appendix S1).

According to Carlton (1999), a wide variety of mollusk species have had their native geographic ranges reshaped by human activity, making their exact native ranges difficult to identify. This was reflected in the fact that many of the native ranges identified for a species were not identical between different sources. This was not problematic when applying EICAT scores because there was no case where impacts were recorded in areas that may be deemed dubiously native. However, to obtain native range latitude data, it was necessary to explicitly identify the native range of each species. Hence the most inclusive native range identified was taken as the “true” native range, with the intention of maximizing overlap with its veritable indigenous distribution. The explicit geographic borders of these ranges were identified in accordance with various information sources (provided in Supporting Information Appendix S1). To obtain native range latitude data, the Global Biodiversity Information Facility (GBIF) database (https://www.gbif.org/) was used. This was done by calculating the difference between the highest and lowest decimal latitude occurrences in the native range for each species.

### 2.2 Analyses

For the analyses, all DD entries were removed from the dataset and the five impact levels for both schemes converted into numeric variables, with numbers 1 to 5 corresponding to the five impact categories (e.g., 1 for MC and 5 for MV respectively; as per Kumschick et al., 2017). To assess whether environmental and socioeconomic impacts were correlated, Kendall's tau correlation test was conducted to allow for comparison of ordinal responses. We separately analyzed maximum impacts across all records to account for the “worst case scenario” and median impacts to account for the range in impacts recorded.

To compare impact magnitude among mechanisms and habitat types respectively, Kruskal–Wallis rank sum tests were performed using all impacts recorded in the study for the former, and all records for which habitats were identified for the latter. We excluded mechanisms and habitats with less than seven impact records. Additionally, we performed pairwise comparisons between mechanisms post hoc using a Dunn's test, with *p*‐values adjusted using the Benjamini–Hochberg method (Benjamini & Hochberg, 1995). These analyses could not be conducted for SEICAT scores due to limited data availability.

Kendall's tau correlation tests were conducted to assess the relationships between fecundity and environmental and socioeconomic impacts respectively. The same correlations with impacts were undertaken with native range latitude. We again used both, maximum and median impact scores (see above). Due to fecundity values spanning two orders of magnitude, they were log transformed for analysis. Kendall's tau correlation tests were further used to assess the relationship between EICAT and SEICAT maximum scores and the number of publications found per species, respectively, to test if species which are better‐studied record higher impacts (cf. Kumschick et al., 2017). All analyses were performed in R (version 3.2.1; R Core Team 2015).

## 3 RESULTS

A total of 26 species were assigned EICAT scores and 22 species were assigned SEICAT scores, with the remaining species being DD (Table 1). The highest impacting species under EICAT were *Oxychilus draparnaudi* (predation)*, Helisoma duryi, Tarebia granifera,* and *Theba pisana* (all competition)*,* all recording MR impacts. For SEICAT, the highest impact was MO, recorded by *Theba pisana, Cochlicella barbara,* and *Eobania vermiculata* all affecting material and immaterial goods for good life. Of the evaluated species, 20 were assigned scores under both schemes. Environmental and socioeconomic impacts were not correlated for neither maximum (Kendall's tau = 0.35; *P *=* *0.093) nor median (Kendall's tau = 0.06; *p *=* *0.765) scores.

**Figure 1:**
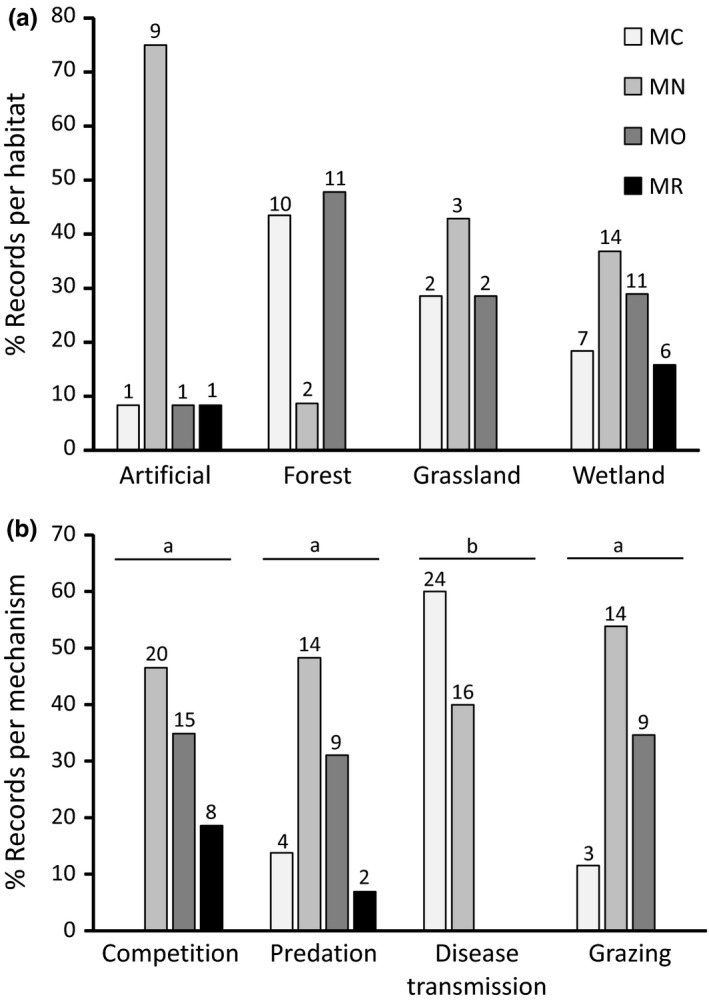
Distribution of impact records and severity across (a) habitats and (b) mechanisms. Actual number of records indicated in small numbers on each bar. Differences in impact magnitude were analyzed using a Kruskal–Wallis test (see main text for results), and letters indicate significant differences in impact magnitude as assessed with a Dunn test (competition predation: *Z* = 1.85, *p *=* *0.07; competition disease transmission: *Z* = 7.11, *p *<* *0.001; predation disease transmission: *Z* = 4.59, *p *<* *0.001; competition grazing: *Z* = 2.01, *p *=* *0.06; predation grazing: *Z* = 0.20, *p *=* *0.84; disease transmission grazing: *Z* = 7.11, *p *<* *0.001)

Environmental impact magnitude did not differ among habitats (Kruskal–Wallis; *χ* ^2^ = 2.39; *df* = 3; *p *=* *0.49). The most common habitat in which gastropod impacts were recorded was wetlands, with 38 records, as well as having the highest number of upper tier impacts (MO or higher; Figure 1a). Environmental impacts were significantly different among mechanisms (Kruskal–Wallis; *χ* ^2^ = 53.30; *df* = 3; *p *<* *0.001). The post hoc test showed that impacts under the “transmission of diseases” mechanism were significantly lower than those recorded under any other mechanism tested, and there was a trend toward competition impacts being larger than the other mechanisms (Figure 1b). Competition was found to be the most common mechanism by which gastropods cause impact, as well as having the highest number of upper tier impacts, more than double that of the second highest mechanism (Figure 1b).

The most commonly impacted habitat regarding socioeconomic impact was “Artificial – Terrestrial” with 140 observations, representing 94.6% of socioeconomic impacts for which habitats were identified. This was the only habitat with upper tier socioeconomic impacts. In comparison with environmental impacts, all habitats recorded upper tier impacts, with the most severe environmental impact in the “Artificial – Terrestrial” habitat being MR.

Data on fecundity and native range latitude were available for 11 species and 25 species respectively. Fecundity did not correlate with maximum environmental (Kendall's tau = −0.38; *p *=* *0.152) or socioeconomic impact (Kendall's tau = 0.36; *p = *0.217), nor with median impacts (environmental: Kendall's tau = −0.33; *p *=* *0.224; socioeconomic: Kendall's tau = −0.04; *p *=* *0.9). Similar results were found for native range latitude: neither maximum environmental (Kendall's tau = −0.14; *p *=* *0.397) or socioeconomic impact (Kendall's tau = −0.26; *p *=* *0.152), nor median impacts (environmental: Kendall's tau = −0.23; *p *=* *0.18; socioeconomic: Kendall's tau = −0.32; *p *=* *0.09) were correlated with it.

Higher impacting species were found to have a higher number of publications underpinning their impacts (EICAT: Kendall's tau = 0.60; *p *= <0.001; SEICAT: Kendall's tau = 0.38*; p *=* *0.035). For EICAT, this remained significant after removing a high‐leverage observation, *Tarebia granifera* (Kendall's tau = 0.56; *p *= <0.001).

## 4 DISCUSSION

This study has successfully categorized the global impacts of South African alien gastropods into EICAT and SEICAT, adding a novel taxonomic group to a growing list of universally comparable alien impact assessments (Bacher et al., 2017; Evans et al., 2016; Kumschick et al., 2017). Information on socioeconomic and/or environmental impact was available for most species included in this study (82.4%), which is comparable to other taxa (arthropods: 77.9%; birds: 84.6%; fish: 91.4%; mammals: 97%; plants: 93.8% Kumschick et al., 2015; but see Measey et al., 2016 for amphibians: 41%). This indicates that gastropod impacts are well researched, further reinforced by a high average number of publications per studied species for environmental (5.6) and socioeconomic (8.9) impacts (Bacher et al., 2017; Evans et al., 2016). The fact that gastropods’ socioeconomic impacts are better‐studied than their environmental impacts is likely due to their global status as agricultural and horticultural pests (here included under “Material and immaterial goods for good life”; Barker, 2002; Hollingsworth & Armstrong, 2003; Keiser, Häberli, & Stamp, 2012; Nash & Hoffmann, 2012; Simms, Ester, & Wilson, 2006), which provides economic incentive to study them. This is reflected in the vast amount of research effort put into mitigating agricultural and horticultural impacts by gastropods (Charwat et al., 1995; Coupland & Baker, 1995; Desbiolles, Ballantyne, & Richards, 2003; Jeong, Lee, Hong, Shin, & Yun, 2012; Nash & Hoffmann, 2012). An example is a research program in Australia, supported by the Australian grain industry in 2000. This aimed solely at modifying existing crop harvesting technology to reduce snail contamination, namely by *Cochlicella barbara* and *Theba pisana,* to harvested grain (MO impact: Coupland & Baker, 1995; Desbiolles et al., 2003). Indeed, agricultural and horticultural impacts are the most common means by which gastropods harm society, representing 70% of SEICAT scores. However, these impacts are generally relatively low only affecting individual persons rather than societal structure at large (Table 1).

A potential explanation for the relatively low impacts recorded is that artificial socioeconomic systems may be more resilient to impact than environmental systems. Cities, for example, have been described as extraordinarily resilient to change (Allenby & Fink, 2005; Fiksel, 2006). Socioeconomic resilience has been described as taking effect at the community level, such that socioeconomic systems remain inert despite individual people making up these systems experiencing change (Adger, 2000). For example, an agricultural corporation that oversees many farms may experience harvesting problems and yield losses due to gastropod contamination, which would result in difficulties experienced by laborers in carrying out their jobs and potentially experience income reductions (MN impact). However, due to the aggressive response of agricultural science and management in researching and implementing solutions, gastropod contamination would very seldom escalate to a point whereby liquidation or abandonment of farms is necessary (MO or higher impact; e.g., Charwat et al., 1995; Fabian et al., 2012; Hollingsworth, Follett, & Armstrong, 2003; Prystupa, Holliday, & Webster, 1987; Simms et al., 2006; Wilson, Hughes, Hamacher, Barahona, & Glen, 1996). This description of socioeconomic resilience is reflected by the high number of impacts on the well‐being of individuals (MN), but very few resulting in declines or abandonment of human activities (MO or higher). Relatively low socioeconomic impacts have also been recorded for mammals (Hagen & Kumschick, 2018) and amphibians (Bacher et al., 2017), except in two cases where cultural practices of indigenous communities were detrimentally affected by the alien species (dogs contributing to decline of vultures, which affects burial rituals: Prakash et al., 2003; cane toads affecting bush tucker hunting in Australia: Van Dam, Walden, & Begg, 2002). On that note, it is important to state that our study only shows evidence of impact in developed countries due to lacking records from less affluent regions, and it is unclear if low impacts to agriculture is a general rule in developing countries with less scientific and managerial resources. Despite South Africa being a developing country, it has a well‐developed agricultural sector, and recorded only MN impacts to agriculture in this study (e.g., Herbert, 1997), and showed an aggressive management response to gastropod pests (Herbert & Sirgel, 2001).

Environmental impacts on the other hand are severe for many species, and the absence of a correlation between SEICAT and EICAT scores might indicate they are generally of greater magnitude than socioeconomic impacts. However, comparing the two measures directly assumes scale equivalence between the two schemes, and even though the two systems follow the same general structure and share many common traits (i.e., nonlinear impact levels based on orders of magnitude, a common currency each throughout the impact levels) it needs to be further evaluated whether the same impact levels should be seen as equal. We encourage a more thorough discussion of the issue of scale equivalence as it would greatly benefit the prioritization process for resource allocation and management, but this is beyond the scope of this study.

It is generally well known that aliens can inflict enormous environmental impacts (Blackburn, Cassey, Duncan, Evans, & Gaston, 2004; Karatayev, Burlakova, Karatayev, & Padilla, 2009; Mack et al., 2000; Pimentel, Zuniga, & Morrison, 2005; Savidge, 1987). Gastropods are no exception to this, with many species in this study recording high impacts involving local extinctions. *Theba pisana,* and *Helisoma duryi* were implicated in causing MR impacts to beach‐dwelling snails and snails inhabiting artificial drains respectively (Christie et al., 1981; Rumi et al., 2010). Moreover, various gastropods have been consistently related to declines in endemic island snail fauna populations, namely in Hawai'i, New Zealand and the Canary Islands (Curry & Yeung, 2013; Curry et al., 2016; Kappes et al., 2009; Mahlfeld, 2000; Meyer & Cowie, 2010). This is potentially due to the uniqueness and vulnerability of island habitats (Mueller‐Dombois & Loope, 1990). One gastropod, *Tarebia granifera,* was implicated in causing local extinctions (MR) to native snails in wetlands in Puerto Rico (Giboda, Malek, & Correa, 1997), Venezuela (Pointier & Giboda, 1999), South Africa (De Kock & Wolmarans, 2008; Miranda & Perissinotto, 2014), and Cuba (Karatayev et al., 2009).

South Africa may be particularly vulnerable to high impacts by *Tarebia granifera*, as it was the only species to record upper tier impacts in this country, with several pieces of evidence showing declines to native populations (e.g., Jones et al., 2017; Miranda & Perissinotto, 2012; Miranda, Perissinotto, & Appleton, 2011). No other continental country recorded this extent of upper tier impacts by *Tarebia granifera*—only Venezuela recorded a single MR impact (Pointier & Giboda, 1999). It evidently represents a severe threat to South African native wetland invertebrate fauna, as it has shown to be for many island nations’ wetlands. South Africa may not have the managerial resources to deal with an invader that is geographically widespread over the eastern and northern parts of the country (Appleton, Forbes, & Demetriades, 2009), so it is probable that this threat may persist into the foreseeable future. Our data show that *Tarebia granifera* is a major threat to native wetland fauna globally. Even though we found no difference in impact magnitude between habitats, wetlands were the most commonly impacted and had the most upper tier impacts (Figure 1a), suggesting that gastropods are most likely to cause severe impacts within this habitat. This reinforces wetland habitats status as a global conservation priority (Dawson, Berry, & Kampa, 2003).

Despite gastropods showing no severe impacts via disease transmission (Figure 1b), one species, *Zonitoides arboreus,* has been shown to be an intermediate host of a disease that has caused deaths of captive species (classified as a socioeconomic impact; Walden et al., 2017). This suggests that the potential for higher tier impacts via disease transmission is evident in the gastropods. Similarly, gastropods were found to be agents of disease transmission to humans (Ash, 1976; Boray, 1978; Kim, Hayes, Yeung, & Cowie, 2014; Senanayake et al., 2003), the most prominent disease being eosinophilic meningitis, caused by infection with *Angiostrongylus cantonensis,* a parasitic nematode of which various gastropods are intermediate hosts. Schistosomiasis was also prominent in this study, known to be a highly pathogenic human disease that can result in mortality (Burke et al., 2009). However, the studies describing human death did not specify particular gastropod species that vector the disease; therefore, these could not be assessed under SEICAT. As such, none of the impacts on human health were upper tier impacts (SEICAT classifies human death as MO impact). This raises potential concerns regarding the classification of impacts via disease transmission (see also Measey et al., 2016), and it should be further explored how the direct impact of the disease can be disentangled from the impact caused by its transmission.

It was estimated in 1989 that more than 4 million people were infected with schistosomiasis in South Africa (Utroska et al., 1990). Given that attempted control programs have been largely unsuccessful (Magaisa, Taylor, Kjetland, & Naidoo, 2015), this figure is likely an underestimate for the current situation. It is likely that the alien gastropods that were reported as vectors of the disease in this study (*Aplexa marmorata* and *Radix rubiginosa*), and which both occur in South African schistosomiasis risk areas (Appleton & Miranda, 2015; Magaisa et al., 2015), play a role in transmission to humans in South Africa, despite no disease transmission socioeconomic impacts being recorded there. *Angiostrongylus cantonensis* has been found in South Africa in rats in KwaZulu‐Natal (Archer, Appleton, Mukaratirwa, & Hope, 2011). Despite a lack of evidence of infection in humans in South Africa, it is likely, as in other Southern Hemisphere countries (e.g., Brazil, Australia), multiple cases of human infection transmitted by South African alien gastropods were recorded in this study (Caldeira et al., 2007; Morassutti, Thiengo, Fernandez, Sawanyawisuth, & Graeff‐Teixeira, 2014; Rambo, Agostini, & Graeff‐Teixeira, 1997; Senanayake et al., 2003). Furthermore, there are multiple species alien to South Africa, all which occur in KwaZulu‐Natal (Appleton & Miranda, 2015; Herbert, 2010), that have been shown to vector the disease (*Cornu aspersum, Limax maximus, Bradybaena similaris, Deroceras laeve, Physa acuta, Limacus flavus, Zonitoides arboreus, Deroceras reticulatum, Lehmannia valentiana,* and *Oxychilus alliarius*). It is unclear to what degree these alien gastropods may pose a disease threat to humans in South Africa, and if their eradication would benefit public health, as other native or non‐native species may simply provide vectors. Further research into this issue is needed. Nonetheless, it is unlikely that developing countries with a lack of management resources and other urgent socioeconomic problems will have the capacity to manage the multiple alien gastropods that pose a disease threat to humans.

On another note, the fact that competition was shown to be the most common mechanism by which gastropods cause impacts, as well as having the highest number of severe impacts suggests that alien gastropods are more likely to cause significant harm to native gastropods than to other taxa.

It is of interest that gastropods and birds, two very distant taxonomic groups, converge on similar probabilities (~0.3) of causing upper tier (MO or higher) environmental impacts overall (Evans et al., 2016). This raises the question whether the proportion of severe impacts caused by harmful alien species globally is the same across taxa. Should this be supported by further research, it may provide scope for universal alien impact predictions, which will be significant for various risk assessment approaches (Keller & Kumschick, 2017). This highlights the need for further application of EICAT to different taxonomic groups.

Our results do not support the general importance ascribed to gastropod fecundity for their impacts (Barker, 1991; De Kock & Wolmarans, 2008; Keller et al., 2007; Rumi et al., 2010). Rumi et al. (2010) describe a local extinction (MR) caused by *Theba pisana* as a result of its high reproductive rate (having the second highest fecundity, >1,500 eggs/female/year, Supporting Information Table S1), however the species with the highest fecundity, *Physa acuta* (>2,500) only reached a score of MN (Table 1). These conflicting results may be due to the context dependency of alien introductions. For example, gastropod fecundities have been shown to fluctuate with temperature and moisture (Brackenbury & Appleton, 1991; Hadfield, 1989; Kozlowski, 2000; Nash & Hoffmann, 2012), and perhaps these species have not found habitats conducive to maximum reproductive output. Given the limited availability of fecundity information (11 values), it would be interesting to test if this, and the absence of any correlations with impact, might change with more data.

The absence of a correlation between native range latitude and environmental impacts is surprising, being contrary to the findings for birds and mammals alien to Europe (Kumschick et al., 2013), and cactaceae (Novoa et al., 2016). Given the complex nature of alien impact (Simberloff et al., 2013) there is reason to believe that native range latitude indeed has no relation to impact, at least for gastropods. A good example of this is the genus *Oxychilus*, whose species have narrow native range latitudes, but have caused significant declines to the Hawai'ian snail fauna (Curry & Yeung, 2013; Curry et al., 2016; Meyer & Cowie, 2010). The difficulty in identifying true native distributions due to human influence (Carlton, 1999) may be confounded with the native range latitude correlations. For example, *Helisoma duryi* had one native range indicated as Florida (Madsen, 1985) and another as North America (Madsen & Frandsen, 1989). Using a more inclusive approach, the native range sizes of this and other species may have been inflated.

The fact that environmental and socioeconomic impact magnitudes were positively correlated to the number of publications suggests one of two scenarios: First, there might be a publication bias, and the maximum scores for species that are underrepresented in the literature are not a true reflection of their impact. Second, research may focus on species that have greater impacts, therefore research effort reflects a species true impact. The latter scenario is supported by Pyšek et al. (2008), showing that research tends to focus on species that are the most relevant to the environment/society. This probably applies to this study, due to the species being on average well represented in the literature (Bacher et al., 2017; Evans et al., 2016). Moreover, socioeconomic impacts are particularly likely to have been noticed, because humans are directly affected.

Generally, EICAT and SEICAT scores could be derived from all impact records found for gastropods, which shows the applicability of the scoring schemes across taxa, especially given this was only the third application of SEICAT following amphibians (Bacher et al., 2017) and some mammals (Hagen & Kumschick, 2018). However, the scoring schemes may have room for improvement. For example, one score recorded for *Tarebia granifera* was described as a trophic cascade (Hill, Jones, Hill, & Weyl, 2015), and there was no mechanism to describe this under EICAT. This was assigned the “structural impact on ecosystems” mechanism. Furthermore, EICAT methodology dictates that maximum scores are the only indication of the severity of impact. We additionally used median values for our analyses to account for the range in impact magnitudes recorded and suggest that each study should assess which measure is most suitable for its purpose. Furthermore, we suggest that EICAT and SEICAT should consider incorporating the likelihood of a high score being realized into the overall quantification of the impact of a species. Despite these limitations, the analyses undertaken in this study shed light on the nature and context dependency of gastropod impacts. This has great relevance to many risk assessment approaches, which can ultimately aid conservation management. Further studies should apply these schemes to multiple novel taxa, allowing for potential generic patterns to be identified, which would greatly improve the understanding of alien impacts in an ever‐changing biological realm.

## CONFLICT OF INTEREST

None declared.

## AUTHORS’ CONTRIBUTION

Both authors conceptualized the study and revised the manuscript. DK collected and analyzed the data and led the writing of the manuscript with inputs from SK.

## DATA ACCESSIBILITY

All data used in this study are available in the main manuscript or attached in the Supporting Information.
