# Supplementary material for: Gastropods alien to South Africa cause severe environmental harm in their global alien ranges across habitats
Source: Ecol Evol. 2018 Jul 22;8(16):8273–85. doi: 10.1002/ece3.4385 (PMC6144998; doi:10.1002/ece3.4385)
Supplement: Supplementary file 1 [file ECE3-8-8273-s001.docx]

**Supporting Information**

**Table S1:** Trait variables for all 34 species included in this study. Data only shown if available. Native range latitudes are in decimal degrees.

| **Species** | **Native range** | **Native range latitude** | **Fecundity [eggs/female/year]** | |
| --- | --- | --- | --- | --- |
| *Aplexa marmorata* | South America | | |  |
| *Arion hortensis* | Western and southern Europe | 15.15 | |  |
| *Arion intermedius* | Central and western Europe | 12.69 | |  |
| *Bradybaena similaris* | southeast Asia and Indonesia | 25.00 | |  |
|  |  |  | |  |
| *Cochlicella barbara* | Mediterranean | 12.29 | |  |
| *Cochlicopa cf. lubrica* | Europe, Northern America |  | |  |
| *Cochlicopa cf. lubricella* | Europe, Asia, North America |  | |  |
|  |  |  | |  |
| *Cornu aspersum* | southern and western Europe | 24.32 | | 430 |
| *Deroceras laeve* | Holarctic (high latitudes) | 44.08 | | 612 |
| *Deroceras panormitanum* | Mediterranean | 3.97 | | 612 |
| *Deroceras reticulatum* | Europe | 29.54 | | 612 |
| *Discus rotundatus* | Europe |  | |  |
| *Eobania vermiculata* | Mediterranean region | 14.74 | |  |
| *Gyraulus chinensis* | South East Asia |  | |  |
| *Helisoma duryi* | North America | 11.71 | | 100 |
| *Lauria cylindracea* | Western Europe, Mediterranean |  | |  |
| *Lehmannia nyctelia* | N Africa, Central and SE Europe | | | |
| *Lehmannia valentiana* | Iberian Peninsula | 2.98 | |  |
| *Limacus flavus* | Europe | 23.16 | |  |
| *Limax maximus* | Europe, North Africa, and Asia minor (western palearctic) | 38.40 | | 223 |
| *Littorina saxatilis* | Eastern North Atlantic | 19.59 | |  |
| *Lymnaea columella* | North America | 29.33 | | 619 |
| *Milax gagates* | Mediterranean | 0.60 | | 100 |
| *Oxychilus alliarius* | Western Europe | 15.18 | |  |
| *Oxychilus cellarius* | Western Europe | 10.77 | |  |
| *Oxychilus draparnaudi* | Europe, North Africa, and Asia minor | 35.78 | |  |
| *Physa acuta* | North America | 30.29 | | 2598 |
| *Radix rubiginosa* | Indo-China and Indonesia | 27.33 | |  |
| *Tarebia granifera* | India, Southeast Asia, the Philippines, Japan. Hawaii | 30.62 | | 213 |
| *Thais blanfordi* | Tropical Indo-Pacific |  | |  |
| *Theba pisana* | Mediterranean, western European and north African coastal distribution | 27.49 | | 1525 |
| *Vallonia costata* | Europe, Eastern USA | | | |
| *Vallonia pulchella* | Holarctic (high latitudes) | 45.00 | |  |
| *Zonitoides arboreus* | Canada, Central America, Caribbean | 46.15 | |  |

**Appendix S1:** Literature sources for native range and fecundity information

Abbott, R. T. (1952). A study of an intermediate snail host (*Thiara granifera*) of the oriental lung fluke (Paragonimus). *Proceedings of the United States National Museum*, 102, 71-116.

Albrecht, C. et al., (2014). Invaders versus endemics: Alien gastropod species in ancient Lake Ohrid. *Hydrobiologia*, 739, 163-174.

Baily, J. L. (1931). Some data on growth, longevity, and fecundity in *Lymnaea columella* Say. *Biologia Generalis*, 7, 407-428.

Barker, G. M. (1999). Naturalised terrestrial Stylommatophora (Mollusca: Gastropoda). *Fauna of New Zealand*, 38, 1-253.

Boerger, H. (1975). A comparison of the life cycles, reproductive ecologies, and size–weight relationships of *Helisoma anceps*, *H. campanulatum*, and *H. trivolvis* (Gastropoda, Planorbidae). *Canadian Journal of Zoology*, 53, 1812-1824.

Brackenbury, T. D., & Appleton, C. C. (1991). Effect of controlled temperatures on gametogenesis in the gastropods *Physa acuta* (Physidae) and *Bulinus tropicus* (Planorbidae). *Journal of Molluscan Studies*, 57, 461-469.

Central Intelligence Agency, (2009, May). The World Factbook: Field listing – Location. Retrieved from https://www.cia.gov/library/publications/the-world-factbook/fields/2144.html.

Cowie, R. H. (1990). Climatic selection on body colour in the land snail *Theba pisana* (Pulmonata: Helicidae). *Heredity*, 65, 123-126.

European Union, (2017). http://eurovoc.europa.eu/.

Fanjul, E. A., Gómez, B. P., & Sánchez-Arévalo, I. R. (1997). A description of the tides in the Eastern North Atlantic. *Progress in Oceanography*, 40, 217-244.

Herbert, D. G. (2010). *The introduced terrestrial molluscs of South Africa.* Pretoria, South Africa: SANBI.

Hubendick, B. (1951). Recent Lymnaeidae. Their variation, morphology, taxonomy, nomenclature, and distribution. *The text*, 3, 1–223.

Keller, R. P., Drake, J. M., & Lodge, D. M. (2007). Fecundity as a basis for risk assessment of nonindigenous freshwater molluscs. *Conservation Biology*, 21, 191-200.

Kerney, M. P., Cameron, R. A., & Jungbluth, J. H. (1983). *Die Landschnecken Nord-und Mitteleuropas*. Hamburg; Berlin, Germany: Parey.

Lovett, A.L., & Black, A.B. (1920). The gray garden slug: With notes on allied forms. *Station Bulletin*, 170, 1-43.

Madsen, H., & Frandsen, F. (1989). The spread of freshwater snails including those of medical and veterinary importance. *Acta tropica*, 46, 139-146.

Mead, A. et al., (2011). Introduced and cryptogenic marine and estuarine species of South Africa. *Journal of Natural History*, 45, 2463-2524. doi:10.1080/00222933.2011.595836

Meyer, W. M., & Cowie, R. H. (2010). Invasive temperate species are a threat to tropical island biodiversity. *Biotropica*, *42*(6), 732-738. doi:10.1111/j.1744-7429.2010.00629.x

Nash, M. A., & Hoffmann, A. A. (2012). Effective invertebrate pest management in dryland cropping in southern Australia: The challenge of marginality. *Crop protection*, 42, 289-304. doi:http:/dx.doi.org/10.1016/j.cropro.2012.06.017

Oteros, J. (2014). *Modelización del ciclo fenológico reproductor del olivo* (Doctoral dissertation). University of Córdoba, Córdoba, Spain.

Picker, M. D., & Griffiths, C. L. (2017). Alien animals in South Africa–composition, introduction history, origins and distribution patterns. *Bothalia-African Biodiversity & Conservation*, 47, 1-19.

Pilsbry, H. A. (1946). Land mollusca of North America (North of Mexico), Vol II: Part 1. [Monographs]. *Academy of Natural Sciences of Philadelphia*, 480-483.

Roth, B., & Sadeghian, P. S. (2006). *Checklist of the land snails and slugs of California*. California, CA: Santa Barbara Museum of Natural History.

Snow, D. W., & Perrins, C. M. (1998). *The Birds of the Western Palearctic*. Oxford, UK: Oxford University Press.

Solem, A. (1964). New records of New Caledonian nonmarine mollusks and an analysis of the introduced mollusks. *Pacific Science,* 8, 130-137.

United Nations Statistics Division, (2015, May). Standard Country and Area Codes Classifications (M49). Retrieved from https://unstats.un.org/unsd/methodology/m49/.

Waldén, H. W. (1961). On the variation, nomenclature, distribution and taxonomic position of *Limax* (Lehmannia) *valentianus* FÉRUSSAC (Gastropoda: Pulmonata). *Arkiv för Zoologi*, 15, 71-96.

Wiktor, A. (1987). *Milacidae (Gastropoda, Pulmonata): Systematic monograph*. Warsaw, Poland: Państwowe Wydawnictwo Naukowe.

Wiktor, A. (2001). *Deroceras* (Deroceras) *panormitanum* (Lessona et Pollonera, 1882)-a new introduced slug species in Poland (Gastropoda: Pulmonata: Agriolimacidae). *Folia Malacologica*, 9. 155-157.

**Appendix S2:** Literature used to score gastropod environmental impacts under EICAT

Abdallah, A., & Nasr, T. (1973). *Helisoma H. duryi* as a means of biological control of schistosomiasis vector snails. *Journal of the Egyptian Medical Association*, 56, 514-520.

Aghazadeh, M. et al., (2015). A survey of *Angiostrongylus* species in definitive hosts in Queensland. *International Journal for Parasitology: Parasites and Wildlife*, 4, 323-328. doi:http://dx.doi.org/10.1016/j.ijppaw.2015.06.003

Appleton, C., & Miranda, N. (2015). Locating bilharzia transmission sites in South Africa: Guidelines for public health personnel. *Southern African Journal of Infectious Diseases*, 30(3), 95-102. doi:10.1080/23120053.2015.1074438

Ayad, N. et al., (1970). A preliminary study on biological control of the snail intermediate hosts of schistosomiasis in U.A.R. by *Helisoma duryi* snails. *Hydrobiologia.* 35, 196-197.

Barker, G. M. (2002). Gastropods as Pests in New Zealand Pastoral Agriculture, with Emphasis on Agriolimacidae, Arionidae and Milacidae. In G. M. Barker (Ed.), *Molluscs as crop pests* (pp. 361-423). Wallingford, UK: CAB International.

Basch, P. F. (1965). Completion of the life cycle of *Eurytrema pancreaticum* (Trematoda: Dicrocoeliidae). *The Journal of Parasitology*, 51, 350-355.

Brooker, B. (2016). *Distribution and predictors of non-indigenous marine species within South Africa's MPA network* (Unpublished doctoral dissertation). University of Cape Town, Cape Town, South Africa.

Bunnag, T. et al., (1983). *Schistosoma incognitum* and its zoonotic potential role in Phitsanulok and Phichit provinces, northern Thailand. *The Southeast Asian Journal of Tropical Medicine and Public Health*, 14, 163-170.

Butcher, A. R., & Grove, D. I. (2001). Description of the life-cycle stages of *Brachylaima cribbi* n. sp. (Digenea: Brachylaimidae) derived from eggs recovered from human faeces in Australia. *Systematic Parasitology*, 49, 211-221.

Butcher, A. R., & Grove, D. I. (2003). Field prevalence and laboratory susceptibility of southern Australian land snails to *Brachylaima cribbi* sporocyst infection. *Parasite,* 10, 119-125. doi:http://dx.doi.org/10.1051/parasite/2003102119

Butcher, A. R., & Grove, D. I. (2005). Second intermediate host land snails and definitive host animals of *Brachylaima cribbi* in southern Australia. *Parasite*, 12, 31-37. doi:http://dx.doi.org/10.1051/parasite/2005121031

Butler, J. M. et al., (1980). Displacement of a colony of *Biomphalaria glabrata* by an invading population of *Tarebia granifera* in a small stream in Puerto Rico. *Caribbean Journal of Science*, 16, 73-79.

Cadiz, F. J., & Gallardo, C. S. (2007). *Arion intermedius* (Gastropoda: Stylommatophora); first record of this introduced slug in Chile, with notes on its anatomy and natural history. *Revista Chilena de Historia Natural*, 80(1), 99-108.

Cañete, R. et al., (2004). Population dynamics of intermediate snail hosts of *Fasciola hepatica* and some environmental factors in San Juan y Martinez municipality, Cuba. *Memórias do Instituto Oswaldo Cruz*, 99, 257-262.

Carlton, J. T., & Cohen, A. N. (1998). Periwinkle's progress: The Atlantic snail *Littorina saxatilis* (Mollusca: Gastropoda) establishes a colony on a Pacific shore. *Veliger,* 41, 333-338.

Carney, W. P. et al., (1977). *Schistosoma incognitum* from Cikurai, West Java, Indonesia. *International Journal for Parasitology*, 7, 361-366.

Christie, J. D. et al., (1981). Interactions between St. Lucian *Biomphalaria glabrata* and *Helisoma duryi*, a possible competitor snail, in a semi-natural habitat. *Acta Tropica*, 38, 395-417.

Cowie, R. H. (2001). Can snails ever be effective and safe biocontrol agents?. *International Journal of Pest Management*, 47, 23-40.

Cribb, T. H. (1990). Introduction of a *Brachylaima* species (Digenea: Brachylaimidae) to Australia. *International Journal for Parasitology*, 20, 789-796.

Curry, P. A., & Yeung, N. W. (2013). Predation on endemic Hawaiian land snails by the invasive snail *Oxychilus alliarius*. *Biodiversity and Conservation*, 22, 3165-3169. doi:10.1007/s10531-013-0576-3

Curry, P. A. et al., (2016). Rapid range expansion of an invasive predatory snail, *Oxychilus alliarius* (Miller 1822), and its impact on endemic Hawaiian land snails. *Biological Invasions*, 18, 1769-1780. doi:10.1007/s10530-016-1119-0

Darwall, W. R. T. et al., (2009). *The status and distribution of freshwater biodiversity in Southern Africa.* Gland, Switzerland: IUCN and Grahamstown, South Africa: SAIAB.

De Kock, K. N., & Wolmarans, C. T. (2008). Invasive alien freshwater snail species in the Kruger National Park, South Africa. *Koedoe: African Protected Area Conservation and Science*, 50(1), 49-53.

Faust, E. C., & Bonne, C. (1948). Mammalian blood flukes of Celebes. *The Journal of Parasitology,* 34, 124-131.

Ferguson, F. F., Oliver-Gonzalez, J., & Palmer, J. R. (1958). Potential for biological control of *Australorbis glabratus*, the intermediate host of Puerto Rican schistosomiasis. *The American Journal of Tropical Medicine and Hygiene*, 7, 491-493.

Forsyth, R. G. (1999). Distributions of nine new or little-known exotic land snails in British Columbia. *Canadian Field-Naturalist,* 113, 559-568.

Fox, L., & Landis, B. J. (1973). Notes on the Predaceous Habits of the Gray Field Slug, *Deroceras laeve* 1 2. *Environmental Entomology*, 2, 306-307.

Frandsen, F. (1976). The suppression, by *Helisoma duryi*, of the cercarial production of *Schistosoma mansoni*-infected *Biomphalaria pfeifferi*. *Bulletin of the World Health Organization,* 53, 385-390.

Frandsen, F., & Christensen, N. Ø. (1977). Effect of *Helisoma duryi* on the survival, growth, and cercarial production of *Schistosoma mansoni*-infected *Biomphalaria glabrata*. *Bulletin of the World Health Organization*, 55, 577-580.

Furuichi, S. (2014). Field observation of predation on paper wasp nests by introduced terrestrial slugs. *Insectes Sociaux*, 61, 95-96. doi:10.1007/s00040-013-0329-z

Giboda, M., Malek, E. A., & Correa, R. (1997). Human schistosomiasis in Puerto Rico: Reduced prevalence rate and absence of *Biomphalaria glabrata*. *The American Journal of Tropical Medicine and Hygiene*, 57, 564-568.

Gomez, J. D., Vargas, M., & Malek, E. A. (1990). Biological control of *Biomphalaria glabrata* by *Thiara granifera* under laboratory conditions. *Tropical medicine and parasitology: Official organ of Deutsche Tropenmedizinische Gesellschaft and of Deutsche Gesellschaft fur Technische Zusammenarbeit (GTZ),* 41, 43-45.

Gouyon, P. H., Fort, P. H., & Caraux, G. (1983). Selection of seedlings of *Thymus vulgaris* by grazing slugs. *Journal of Ecology*, 71, 299-306.

Gracenea, M., & González-Moreno, O. (2002). Life cycle of *Brachylaima mascomai* n. sp.(Trematoda: Brachylaimidae), a parasite of rats in the Llobregat Delta (Spain). *Journal of Parasitology*, 88, 124-133. doi:https://doi.org/10.1645/00223395(2002)088[0124:LCOBMN]2.0.CO;2

Gutierrez, A. et al., (1997). Relationship of the prosobranch snails *Pomacea paludosa*, *Tarebia granifera* and *Melanoides tuberculata* with the abiotic environment and freshwater snail diversity in the central region of Cuba. *Malacological Review*, 30, 39-44.

Hickford, M. J., Cagnon, M., & Schiel, D. R. (2010). Predation, vegetation and habitat-specific survival of terrestrial eggs of a diadromous fish, *Galaxias maculatus* (Jenyns, 1842). *Journal of Experimental Marine Biology and Ecology*, 385, 66-72. doi:10.1016/j.jembe.2010.01.010

Hill, J. M. et al., (2015). Comparisons of isotopic niche widths of some invasive and indigenous fauna in a South African river. *Freshwater Biology*, 60, 893-902. doi:10.1111/fwb.12542

Holland, K. D., Mcdonnell, M. J., & Williams, N. S. (2007). Abundance, species richness and feeding preferences of introduced molluscs in native grasslands of Victoria, Australia. *Austral Ecology*, 32, 626-634. doi:10.1111/j.1442-9993.2007.01749.x

Joe, S. M. (2006). *Impact of alien slugs on native plant seedlings in a diverse mesic forest, O ‘ahu, Hawai ‘i, and a study of slug food plant preferences* (Unpublished master’s thesis). University of Hawaii at Manoa, Honolulu, Hawaii.

Joe, S. M., & Daehler, C. C. (2008). Invasive slugs as under-appreciated obstacles to rare plant restoration: Evidence from the Hawaiian Islands. *Biological Invasions*, 10, 245-255. doi:10.1007/s10530-007-9126-9

Jones, R. W. et al., (2017). The abundance of an invasive freshwater snail *Tarebia granifera* (Lamarck, 1822) in the Nseleni River, South Africa. *African Journal of Aquatic Science*, 42, 75-81. doi:10.2989/16085914.2017.1298984

Kappes, H. et al., (2009). Native and introduced gastropods in laurel forests on Tenerife, Canary Islands. *Acta Oecologica*, 35, 581-589. doi:10.1016/j.actao.2009.05.004

Karatayev, A. Y. et al., (2009). Introduction, distribution, spread, and impacts of exotic freshwater gastropods in Texas. *Hydrobiologia*, 619, 181-194. doi:10.1007/s10750-008-9639-y

Kim, J. R. et al., (2014). Diverse gastropod hosts of *Angiostrongylus cantonensis*, the rat lungworm, globally and with a focus on the Hawaiian Islands. *PLoS One*, 9(5), e94969. doi:10.1371/journal.pone.0094969

Kralka, R. A., & Samuel, W. M. (1984). Experimental life cycle of *Protostrongylus boughtoni* (Nematoda: Metastrongyloidea), a lung worm of snowshoe hares, *Lepus americanus*. *Canadian Journal of Zoology*, 62, 473-479.

Lankester, M. W., & Anderson, R. C. (1968). Gastropods as intermediate hosts of *Pneumostrongylus tenuis* Dougherty of white-tailed deer. *Canadian Journal of Zoology*, 46, 373-383.

Lankester, M. W., & Peterson, W. J. (1996). The possible importance of wintering yards in the transmission of *Parelaphostrongylus tenuis* to white-tailed deer and moose. *Journal of Wildlife Diseases*, 32, 31-38. doi:https://doi.org/10.7589/0090-3558-22.4.582

Larned, S. T., Chong, C. T., & Punewai, N. (2001). Detrital fruit processing in a Hawaiian stream ecosystem. *Biotropica*, 33, 241-248.

Lidicker, W. Z. (1976). Experimental manipulation of the timing of reproduction in the California vole*. Researches on Population Ecology*, 18, 14-27.

Lovett, A. L., & Black, A. B. (1920). The Gray Garden Slug: With notes on allied forms. *Station Bulletin*, 170, 1-43.

Mackerras, M. J., & Sandars, D. F. (1955). The life history of the rat lung-worm, *Angiostrongylus cantonensis* (Chen)(Nematoda: Metastrongylidae). *Australian Journal of Zoology*, 3, 1-21.

Madsen, H. (1979). Further laboratory studies on the interspecific competition between *Helisoma duryi* (Wetherby) and the intermediate hosts of *Schistosoma mansoni* Sambon: *Biomphalaria alexandrina* (Ehrenberg) and *B. camerunensis* (Boettger). *Hydrobiologia*, 66, 181-192.

Madsen, H. (1979). Preliminary observation on the role of conditioning and mechanical Interference with egg masses and Juveniles in the competitive relationships Between *Helisoma Duryi* (Wetherby) and the Intermediate host of *Schistosoma Mansoni* Sambon: *Biomphalaria Camerunensis* (Boettger). *Hydrobiologia*, 67, 207-214.

Madsen, H. (1985). The effect of *Helisoma duryi* on the cercarial production of *Schistosoma mansoni*-infected *Biomphalaria alexandrina*: Evaluation of chemical interferences and direct competition. *Parasitology Research*, 71, 71-77.

Madsen, H., & Frandsen, F. (1979). Studies on the interspecific competition between *Helisoma duryi* (Wetherby) and *Biomphalaria camerunensis* (Boettger). Size-weight relationships and laboratory competition experiments. *Hydrobiologia*, 66, 17-23.

Mahlfeld, K. (2000)*. Impact of introduced gastropods on molluscan communities, northern North Island. Conservation Advisory Science Notes* (No. 277). Department of Conservation, Wellington.

Mahlfeld, K. et al., (2012). The conservation status of New Zealand terrestrial Gastropoda excluding Powelliphanta. *New Zealand Entomologist*, 35, 103-109. doi:10.1080/00779962.2012.686313

March, J. G. et al., (2002). Effects of freshwater shrimp assemblages on benthic communities along an altitudinal gradient of a tropical island stream. *Freshwater Biology*, 47, 377-390.

Mead, A. et al., (2011). Introduced and cryptogenic marine and estuarine species of South Africa. *Journal of Natural History*, 45, 2463-2524. doi:10.1080/00222933.2011.595836

Meyer III, W. M., & Cowie, R. H. (2010). Feeding preferences of two predatory snails introduced to Hawaii and their conservation implications. *Malacologia*, 53, 135-144.

Meyer III, W. M., & Yeung, N. W. (2011). Trophic relationships among terrestrial molluscs in a Hawaiian rain forest: Analysis of carbon and nitrogen isotopes. *Journal of Tropical Ecology*, 27, 441-445. doi:10.1017/S02 6646 74110000 5 8

Meyer-Lassen, J., & Madsen, H. (1989). The effect of varying relative density and varying food supply on interspecific competition between *Helisoma duryi* and *Bulinus truncatus* (Gastropoda, Planorbidae). *Journal of Molluscan Studies*, 55, 89-96.

Miranda, N. A. F, & Perissinotto, R. (2012). Stable isotope evidence for dietary overlap between alien and native gastropods in coastal lakes of northern KwaZulu-Natal, South Africa. *PLoS One*, 7(2), e31897. doi:10.1371/journal.pone.0031897

Miranda, N. A. F, & Perissinotto, R. (2014). Benthic assemblages of wetlands invaded by *Tarebia granifera* (Lamarck, 1822)(Caenogastropoda: Thiaridae) in the iSimangaliso Wetland Park, South Africa. *Molluscan Research*, 34, 40-48. doi:http://dx.doi.org/10.1080/13235818.2013.866177

Miranda, N. A. F., & Perissinotto, R. (2014). Effects of an alien invasive gastropod on native benthic assemblages in coastal lakes of the iSimangaliso Wetland Park, South Africa. *African Invertebrates*, 55, 209-228.

Miranda, N. A. F., Perissinotto, R., & Appleton, C.C. (2010). Salinity and temperature tolerance of the invasive freshwater gastropod *Tarebia granifera*. *South African Journal of Science,* 106(3/4), 01-07. doi:10.4102/sajs.v106i3/4.156

Miranda, N. A. F., Perissinotto, R., & Appleton, C.C. (2011). Feeding dynamics of the invasive gastropod *Tarebia granifera* in coastal and estuarine lakes of northern KwaZulu-Natal, South Africa. Estuarine, *Coastal and Shelf Science*, 91, 442-449. doi:10.1016/j.ecss.2010.11.007

Miranda, N. A. F, Perissinotto, R., & Appleton, C.C. (2011). Population structure of an invasive parthenogenetic gastropod in coastal lakes and estuaries of Northern KwaZulu-Natal, South Africa. *PLoS One*, 6(8), e24337. doi:10.1371/journal.pone.0024337

Mitchell, A. J., & Brandt, T. M. (2003, April). Thermal limits of redrimmed melania *Melanoides tuberculata*,(Gastropoda: Prosobranchia: Thiaridae): Implication for control and distribution of a snail that vectors a gill trematode causing serious infection in fish. In *Annual Eastern Fish Health Workshop* (Vol. 52).

Monson, R. A., & Post, G. (1972). Experimental transmission of *Protostrongylus stilesi* to bighorn-mouflon sheep hybrids. *The Journal of Parasitology*, 58, 29-33.

Morgan, J. W. (1997). The effect of grassland gap size on establishment, growth and flowering of the endangered *Rutidosis leptorrhynchoides* (Asteraceae). *Journal of Applied Ecology,* 34, 566-576.

Moslemi, J. M. et al., (2012). Impacts of an invasive snail (*Tarebia granifera*) on nutrient cycling in tropical streams: The role of riparian deforestation in Trinidad, West Indies. *PLoS One*, 7(6), e38806. doi:10.1371/journal.pone.0038806

Mountainspring, S. et al., (1990). Ecology, behavior, and conservation of the Poo-uli (*Melamprosops phaeosoma*). *The Wilson Bulletin*, 102, 109-122.

Nollen, P. M., & Murray, H. D. (1978). *Philophthalmus gralli*: Identification, growth characteristics, and treatment of an oriental eyefluke of birds introduced into the continental United States. *The Journal of Parasitology*, 64, 178-180.

Odendaal, L. J., Haupt, T. M., & Griffiths, C. L. (2008). The alien invasive land snail *Theba pisana* in the West Coast National Park: Is there cause for concern?. *Koedoe*, 50, 93-98.

Perera, A. A. V., & Valderrama, S. P. (2010). Endemic Freshwater molluscs of Cuba and their conservation status. *Tropical Conservation Science*, 3, 190-199.

Perera, D.P.G., Yong, C.M., & Ferrer, L.J. (1991). The biological control of *Fossaria cubensis*, the intermediate host of *Fasciola hepatica*, in 2 localities with different control agents. *Revista Cubana de Medicina Tropical*, 43, 17-20.

Perera, G. et al., (1995). Ecological structure and factors regulating the population dynamics of the freshwater snail populations in Hanabanilla Lake, Cuba. *Malacological Review*, 28, 63-69.

Phillips, C. T., Alexander, M. L., & Howard, R. (2010). Consumption of eggs of the endangered fountain darter (*Etheostoma fonticola*) by native and nonnative snails. *The Southwestern Naturalist*, 55, 115-117.

Pointier, J.P., & Giboda, M. (1999). The case for biological control of snail intermediate hosts of *Schistosoma mansoni*. *Parasitology Today*, 15, 395-397.

Prentice, M.A. (1983). Displacement of *Biomphalaria glabrata* by the snail *Thiara granifera* in field habitats in St. Lucia, West Indies. *Annals of Tropical Medicine & Parasitology*, 77, 51-59.

Raw, J. L., Miranda, N. A. F., & Perissinotto, R. (2013). Chemical cues released by an alien invasive aquatic gastropod drive its invasion success. *PLoS One*, 8(5), e64071. doi:10.1371/journal.pone.0064071

Raw, J. L., Miranda, N. A. F., & Perissinotto, R. (2015). Chemical cues released by heterospecific competitors: Behavioural responses of native and alien invasive aquatic gastropods. *Aquatic Sciences*, 77, 655-666. doi:10.1007/s00027-015-0409-4

Rollo, C.D. (1983). Consequences of competition on the reproduction and mortality of three species of terrestrial slugs. *Researches on Population Ecology*, 25, 20-43.

Rollo, C.D. (1983). Consequences of competition on the time budgets, growth and distributions of three species of terrestrial slugs. *Researches on Population Ecology*, 25, 44-68.

Rondelaud, D. (1977). Résultats et problèmes posés par l'introduction de mollusques Zonitidae dans quelques biotopes à Limnées tronquées en Indre et Haute-Vienne [Results and problems set by the introduction of Zonitidae snails in some biotopes of *Lymnaea trancatula* Müller in Indre and Haute-Vienne, France (author's transl]. *Annales de Parasitologie Humaine et Comparee*, 52, 521-530.

Rondelaud, D. et al., (2006). The control of *Galba truncatula* (Gastropoda: Lymnaeidae) by the terrestrial snail *Zonitoides nitidus* on acid soils. *Biological Control*, 39, 290-299. doi:https://doi.org/10.1016/j.biocontrol.2006.07.015

Rumi, A., Sánchez, J., & Ferrando, N. S. (2010). *Theba pisana* (Müller, 1774) (Gastropoda, Helicidae) and other alien land molluscs species in Argentina. *Biological Invasions*, 12, 2985-2990. doi:10.1007/s10530-010-9715-x

Samson, J., & Holmes, J. C. (1985). Modes of entry of first stage larvae of *Protostrongylus stilesi* and *P. rushi* (Nematoda: Metastrongyloidea) in the snail intermediate host *Vallonia pulchella*. *Canadian Journal of Zoology*, 63, 2481-2482.

Samson, J., & Holmes, J. C. (1985). The effect of temperature on rates of development of larval *Protostrongylus spp*.(Nematoda: Metastrongyloidea) from bighorn sheep, *Ovis canadensis canadensis*, in the snail *Vallonia pulchella*. *Canadian Journal of Zoology*, 63, 1445-1448.

Samuel, W. M., Platt, T. R., & Knispel-Krause, S. M. (1985). Gastropod intermediate hosts and transmission of *Parelaphostrongylus odocoilei*, a muscle-inhabiting nematode of mule deer, *Odocoileus h. hemionus*, in Jasper National Park, Alberta. *Canadian Journal of Zoology*, 63, 928-932.

Shakeel, M. A., & Mowat, D. J. (1992). The potential transmission of clover rot, *Sclerotinia trifoliorum* Erikss., by slugs. *Grass and Forage Science*, 47, 199-202.

Smith, V.R. (2007). Introduced slugs and indigenous caterpillars as facilitators of carbon and nutrient mineralisation on a sub-Antarctic island. *Soil Biology and Biochemistry*, 39, 709-713. doi:10.1016/j.soilbio.2006.09.026

Spratt, D.M., Haycock, P., & Walter, E.L. (2001). Life history and pathogenesis of *Gallegostrongylus australis* (Nematoda: Angiostrongylidae) in Muridae. *Parasite*, 8, 115-125. doi: http://dx.doi.org/10.1051/parasite/2001082115

Stockdale-Walden, H. D. et al., (2015). *Angiostrongylus cantonensis* in introduced gastropods in southern Florida. *Journal of Parasitology*, 101, 156-159.

Upshall, S.M., Burt, M.D.B., & Dilworth, T.G. (1986). *Parelaphostrongylus tenuis* in New Brunswick: The parasite in terrestrial gastropods. *Journal of Wildlife Diseases*, 22, 582-585. doi:https://doi.org/10.7589/0090-3558-22.4.582

Van Oosterhout, C. et al., (2013). Invasive freshwater snails provide resource for native marine hermit crabs. *Aquatic Invasions*, 8, 185-191. doi:http://dx.doi.org/10.3391/ai.2013.8.2.06

Vargas, M., Gomez, J., & Perera, G. (1991). Geographic expansion of *Marisa cornuarietis* and *Tarebia granifera* in the Dominican Republic. *Journal of Medical and Applied Malacology,* 3, 69-72.

Vsevolodov, B.P., & Soboleva, T.N. (1981). Morphobiological characteristics of *Hasstilesia ovis* (Trematoda: Brachylaimidae) and the pathomorphological changes it causes in sheep intestines. *Parazitologiia*, 15, 415-419.

Wardle, D.A., & Barker, G.M. (1997). Competition and herbivory in establishing grassland communities: Implications for plant biomass, species diversity and soil microbial activity. *Oikos,* 80, 470-480.

Wardle, D.A. et al., (1998). Can comparative approaches based on plant ecophysiological traits predict the nature of biotic interactions and individual plant species effects in ecosystems?. *Journal of Ecology*, 86, 405-420.

Zhongzhang, T., & Chongti, T. (1977). The Biology and Epidemiology of *Eurytrema Coelomaticum* (Giard et Billet, 1892) and *Eurytrema Pancreaticum* (Janson, 1889) in Cattle and Sheep in China [J]. *Acta Zoologica Sinica*, 3, 004.

**Appendix S3:** Literature used to score gastropod socio-economic impacts under SEICAT

Abdel-Aty, A. S., & Abdel-Megeed, A. (2014). Molluscicidal activity of Some cyanide derivatives. *Asian Journal of Chemistry*, 26, 7837-7842. doi:http://dx.doi.org/10.14233/ajchem.2014.17982

Abdelgaleil, S. A. (2010). Molluscicidal and insecticidal potential of monoterpenes on the white garden snail, *Theba pisana* (Muller) and the cotton leafworm, *Spodoptera littoralis* (Boisduval). *Applied Entomology and Zoology*, 45, 425-433. doi:10.1303/aez.2010.425

Aghazadeh, M. et al., (2015). A survey of *Angiostrongylus* species in definitive hosts in Queensland. *International Journal for Parasitology: Parasites and Wildlife*, 4, 323-328. doi:http://dx.doi.org/10.1016/j.ijppaw.2015.06.003

Airey, W.J. (1987). Laboratory studies on damage to potato tubers by slugs. *Journal of Molluscan Studies*, 53, 97-104.

Ali, S.M., Yousef, N.M., & Nafady, N.A. (2015). Application of biosynthesized silver nanoparticles for the control of land snail *Eobania vermiculata* and some plant pathogenic fungi. *Journal of Nanomaterials*, 2015, 1-10. doi:http://dx.doi.org/10.1155/2015/218904

Alicata, J. E., & McCarthy, D. D. (1964). On the incidence and distribution of the rat lungworm *Angiostrongylus cantonensis* in the Cook Islands, with observations made in New Zealand and Western Samoa. *Canadian Journal of Zoology*, 42, 605-611.

Altieri, M.A. et al., (1982). Biological control of *Limax maximus* and *Helix aspersa* by indigenous predators in a daisy field in central coastal California. *Acta Oecologica. Oecologia Applicata*, 3, 387-390.

Appleton, C. C., & Nadasan, D. S. (2002). First report of *Tarebia granifera* (Lamarck, 1816)(Gastropoda: Thiaridae) from Africa. *Journal of Molluscan Studies*, 68, 399-402.

Ash, L. R. (1976). Observations on the role of mollusks and planarians in the transmission of *Angiostrongylus cantonensis* infection to man in New Caledonia. *Revista de Biología Tropical*, 24, 163-74.

Baker, G.H. (1989). Damage, population dynamics, movement and control of pest helicid snails in southern Australia. [Monograph]. *British Crop Protection Council*, 175-185.

Baker, G. H. (2008). The population dynamics of the mediterranean snails *Cernuella virgata*, *Cochlicella acuta* (Hygromiidae) and *Theba pisana* (Helicidae) in pasture–cereal rotations in South Australia: A 20-year study. *Australian Journal of Experimental Agriculture*, 48, 1514-1522. doi:https://doi.org/10.1071/EA08031

Baker, G. H., Beckett, S., & Thammavongsa, B. (2012). Are the European snails, *Theba pisana* (Müller, 1774)(Helicidae), *Cernuella virgata* (da Costa, 1778) and *Cochlicella acuta* (Müller, 1774)(Hygromiidae) attracted by potential food odours?. *Crop Protection*, 42, 88-93. doi:https://doi.org/10.1016/j.cropro.2012.05.021

Baker, G. H., & Hawke, B. G. (1990). Life history and population dynamics of *Theba pisana* (Mollusca: Helicidae) in a cereal-pasture rotation. *Journal of Applied Ecology*, 27, 16-29.

Baker, G. H., & Vogelzang, B. K. (1988). Life history, population dynamics and polymorphism of *Theba pisana* (Mollusca: Helicidae) in Australia. *Journal of Applied Ecology*, 25, 867-887.

Baldock, F.C., & Arthur, R.J. (1985). A survey of fascioliasis in beef cattle killed at abattoirs in southern Queensland. *Australian Veterinary Journal*, 62, 324-326.

Banha, F., Marques, M., & Anastácio, P. M. (2014). Dispersal of two freshwater invasive macroinvertebrates, *Procambarus clarkii* and *Physella acuta*, by off‐road vehicles. *Aquatic Conservation: Marine and Freshwater Ecosystems*, 24, 582-591. doi:10.1002/aqc.2453

Barker, G.M. (1989). Slug problems in New Zealand pastoral agriculture. [Monograph]. *British Crop Protection Council*, 59-68.

Barker, G.M. (1991). Slug density‐seedling establishment relationships in a pasture renovated by direct drilling. *Grass and Forage Science*, 46, 113-120.

Barker, G.M. (2002). Gastropods as pests in New Zealand pastoral agriculture, with emphasis on Agriolimacidae, Arionidae and Milacidae. In G.M. Barker (Ed.), *Molluscs as crop pests* (pp. 361-423). Wallingford, UK: CAB International.

Barker, G. M., & Addison, P. J. (1992). Pest status of slugs (Stylommatophora: Mollusca) in two New Zealand pastures. *Crop Protection*, 11, 439-442.

Barker, G. M., & Efford, M. G. (2004). Predatory gastropods as natural enemies of terrestrial gastropods and other invertebrates. In G.M. Barker (Ed.), *Natural enemies of terrestrial molluscs* (pp. 279-403). Wallingford, UK: CAB International.

Barnes, H. F., & Weil, J. W. (1945). Slugs in gardens: Their numbers, activities and distribution. Part 2. *The Journal of Animal Ecology*, 14, 71-105.

Bartsch, P., & Quick, M. E. (1926). An anatomic study of *Zonitoides arboreus* Say. *Journal of Agricultural Research*, 32, 783-791.

Basch, P. F. (1965). Completion of the life cycle of *Eurytrema pancreaticum* (Trematoda: Dicrocoeliidae). *The Journal of Parasitology*, 51, 350-355.

Boray, J. C. (1978). The potential impact of exotic *Lymnaea spp*. on fascioliasis in Australasia. *Veterinary Parasitology*, 4, 127-141.

Boray, J.C., Fraser, G.C., Williams, J.D., & Wilson, J.M. (1985). The occurrence of the snail *Lymnaea columella* on grazing areas in New South Wales and studies on its susceptibility to *Fasciola hepatica*. *Australian Veterinary Journal*, 62, 4-6.

Brooks, A. S. et al., (2005). A laboratory‐based comparison of a molluscicide and an alternative food source (red clover) as means of reducing slug damage to winter wheat. *Pest Management Science*, 61, 715-720. doi:10.1002/ps.1056

Bunnag, T. et al., (1983). *Schistosoma incognitum* and its zoonotic potential role in Phitsanulok and Phichit provinces, northern Thailand. *The Southeast Asian Journal of Tropical Medicine and Public Health*, 14, 163-170.

Butcher, A. R. (2003). *Brachylaima cribbi n. sp.(Digenea: Brachylaimidae): Taxonomy, life-cycle kinetics and infections in animals and humans* (Unpublished doctoral dissertation). The University of Adelaide, Adelaide, Australia.

Butcher, A. R. et al., (2002). *Brachylaima cribbi* (Digenea: Brachylaimidae): Scanning electron microscopical observations of the life-cycle stages. *Journal of Helminthology*, 76, 207-215. doi:10.1079/JOH2002119

Butcher, A. R., & Grove, D. I. (2001). Description of the life-cycle stages of *Brachylaima cribbi* n. sp.(Digenea: Brachylaimidae) derived from eggs recovered from human faeces in Australia. *Systematic Parasitology*, 49, 211-221.

Cabaret, J. (1988). Natural infection of land-snails by protostrongylids on a pasture grazed by sheep in the Rabat area of Morocco. *Veterinary Parasitology*, 26, 297-304.

Cadiz, F. J., & Gallardo, C. S. (2007). *Arion intermedius* (Gastropoda: Stylommatophora); first record of this introduced slug in Chile, with notes on its anatomy and natural history. *Revista Chilena de Historia Natural*, 80(1), 99-108.

Caldeira, R. L. et al., (2007). First record of molluscs naturally infected with *Angiostrongylus cantonensis* (Chen, 1935)(Nematoda: Metastrongylidae) in Brazil. *Memórias do Instituto Oswaldo Cruz*, 102, 887-889. doi:http://dx.doi.org/10.1590/S0074-02762007000700018

Capinera, J. L., & White, J. (2011). *Terrestrial snails affecting plants in Florida* (Publication No*.* EENY-497). Department of Entomology, University of Florida.

Carvalho, O. D. S. et al., (2012). *Angiostrongylus cantonensis* (Nematode: Metastrongyloidea) in molluscs from harbour areas in Brazil. *Memórias do Instituto Oswaldo Cruz*, 107, 740-746. doi:http://dx.doi.org/10.1590/S0074-02762012000600006

Castiellejo, J., Seijas, I., & Villoch, F. (1996, September). Slug and snail pests in Spanish crops and their economical importance. In *Slug & snail pests in agriculture: Proceedings of a Symposium,* British Crop Protection Council, University of Kent, Canterbury, UK.

Chang, C. P. (1990). Evaluation of chemical and exclusion methods for control of *Bradybaena similaris* (Ferussac) on grapevine in Taiwan. *Agriculture, Ecosystems & Environment*, 31, 85-88.

Charlton, J. F. L. (1978). Slugs as a possible cause of establishment failure in pasture legumes oversown in boxes. *New Zealand Journal of Experimental Agriculture*, 6, 313-317.

Charwat, S.M., & Davies, K.A. (1999). Laboratory screening of nematodes isolated from South Australia for potential as biocontrol agents of helicid snails. *Journal of Invertebrate Pathology*, 74, 55-61.

Charwat, S.M., Davies, K.A., & Fraser, H. (1995). *Nematodes as biocontrol agents of helicid snails.* (RIRDC Project No. UA-31A, Publication No. 01/03). Rural Industries Research and Development Corporation.

Clemente, N.L. et al., (2008). Biological studies and phenology of the slug *Deroceras reticulatum* (Müller, 1774)(Pulmonata: Stylommatophora). *Invertebrate Reproduction & Development*, 52, 23-30. doi:10.1080/07924259.2008.9652268

Córdoba, V., & León, S. (2010). Effects of agroecological and conventional handling in slug populations in lettuce in Tenjo, Cundinamarca (Colombia). *Acta Biológica Colombiana*, 15, 115-128.

Coupland, J. B., & Baker, G. (1995). The potential of several species of terrestrial Sciomyzidae as biological control agents of pest helicid snails in Australia. *Crop Protection*. 14, 573-576.

Coupland, J. B., Espiau, A., & Baker, G. (1994). Seasonality, longevity, host choice, and infection efficiency of *Salticella fasciata* (Diptera: Sciomyzidae), a candidate for the biological control of pest helicid snails. *Biological Control*, 4, 32-37.

Davis, A.J. (1989). Effects of soil compaction on damage to wheat seeds by three pest species of slug. *Crop Protection*, 8, 118-121.

Dawkins, G., Luxton, M., & Bishop, C. (1985). Transmission of liquorice rot of carrots by slugs. *Journal of Molluscan Studies*, 51, 83-85.

Desbiolles, J., Ballantyne, T., & Richards, M. (2003). Harvesting snail infested grain crops part I: Snail dislodge bar designs and performance. In G. Quick (Ed.) *International Conference on Crop Harvesting and Processing* (ASAE Publication Number 701P1103e). Louisville, Kentucky USA: American Society of Agricultural and Biological Engineers.

Elliott, L.P. (1969). Certain bacteria, some of medical interest, associated with the slug *Limax maximus*. *Journal of Invertebrate Pathology*, 15, 306-312.

Eshra, E.H. (2014). Toxicity of methomyl, copper hydroxide and urea fertilizer on some land snails. *Annals of Agricultural Sciences*, 59, 281-284. doi:http://dx.doi.org/10.1016/j.aoas.2014.11.017

Fabian, Y. et al., (2012). Diversity protects plant communities against generalist molluscan herbivores. *Ecology and Evolution*, 2(10), 2460-2473. doi:10.1002/ece3.359

Faull, B.W. (1987). Bovine fascioliasis in the Manawatu: Epidemiology and farmer awareness. *New Zealand Veterinary Journal*, 35, 72-74.

Ferguson, C. M., Barratt, B. I. P., & Jones, P. A. (1988). Control of the grey field slug (*Deroceras reticulatum* (Muller)) by stock management prior to direct-drilled pasture establishment. *The Journal of Agricultural Science*, 111, 443-449.

Fox, L., & Landis, B. J. (1973). Notes on the predaceous habits of the gray field slug, *Deroceras laeve* 1 2. *Environmental Entomology*, 2, 306-307.

Gaitán‐Espitia, J. D. et al., (2012). Repeatability of energy metabolism and resistance to dehydration in the invasive slug *Limax maximus*. *Invertebrate Biology*, 131, 11-18.

Glen, D. et al., (2005). Assessing the risk of slug damage to oilseed rape and the need for control measures. *IOBC wprs Bulletin*, 28, 75-78.

Gould, H. J. (1961). Observations on slug damage to winter wheat in East Anglia, 1957–1959. *Plant Pathology*, 10, 142-146.

Gouyon, P.H., Fort, P.H., & Caraux, G. (1983). Selection of seedlings of *Thymus vulgaris* by grazing slugs. *Journal of Ecology*, 71, 299-306.

Grewal, P.S. et al., (2003). Parasitism of molluscs by nematodes: Types of associations and evolutionary trends. *Journal of Nematology*, 35, 146-156.

Guo, Y. H. et al., (2014). Investigation on the species distribution and infection status of host snails of *Angiostrongylus cantonensis* in Shanghai. *Chinese Journal of Parasitology & Parasitic Diseases*, 32, 455-458.

Hammond, R. B. et al., (1999). Slugs in conservation tillage corn and soybeans in the eastern corn belt. *Journal of Entomological Science*, 34, 467-478.

Herbert, D. G. (1997). The terrestrial slugs of KwaZulu-Natal: Diversity, biogeography and conservation (Mollusca: Pulmonata). *Annals of the Natal Museum*, 38, 197-239.

Herbert, D. G., & Sirgel, W.F. (2001). The recent introduction of two potentially pestiferous alien snails into South Africa and the outcomes of different pest management practices: An eradication and a colonization: Research in action. *South African Journal of Science*, 97(7-8), 301-304.

Hollingsworth, R. G., & Armstrong, J. W. (2003). Effectiveness of products containing metaldehyde, copper or extracts of yucca or neem for control of *Zonitoides arboreus* (Say), a snail pest of orchid roots in Hawaii. *International Journal of Pest Management*, 49, 115-122.

Hollingsworth, R.G., Armstrong, J.W., & Campbell, E. (2002). Pest control: Caffeine as a repellent for slugs and snails. *Nature*, 417, 915-916.

Hollingsworth, R. G., Follett, P. A., & Armstrong, J. W. (2003). Effects of irradiation on the reproductive ability of *Zonitoides arboreus*, a snail pest of orchid roots. *Annals of Applied Biology*, 143, 395-399.

Hollingsworth, R.G., & Sewake, K.T. (2002). *The orchid snail as a pest of orchids in Hawaii* (Research Report No. MP-1). University of Hawaii at Manoa, Honolulu, Hawaii: College of Tropical Agriculture and Human Resources.

Horsák, M., Dvořák, L., & Juřičková, L. (2004). Greenhouse gastropods of the Czech Republic: Current stage of research. *Malakológiai Tájékoztató*, 22, 141-147.

Hussein, H. I. et al., (1994). Uscharin, the most potent molluscicidal compound tested against land snails. *Journal of Chemical Ecology*, 20, 135-140.

Idris, A. B., & Abdullah, M. (1997). The phorid fly, *Megaselia scalaris* (Loew), as a candidate for managing molluscicide-resistant round snail, *Bradybaena similaris* (Ferussas). *Resistant Pest Management*, 9, 28-29.

Ingram, J.W. (1931). Soil animals attacking sugar cane. *Journal of Economic Entomology*, 24, 866-869.

Jeong, K. J. et al., (2012). Effective control of slug damage through tobacco extract and caffeine solution in combination with alcohol. *Horticulture, Environment, and Biotechnology*, 53, 123-128. doi:10.1007/s13580-012-0100-9

Kaya, H.K., & Mitani, D.R. (1999). *Molluscicidal nematodes for the biological control of pest slugs* (Slosson Report No. 2000-2001, 1-4).

Keiser, A., Häberli, M., & Stamp, P. (2012). Quality deficiencies on potato (*Solanum tuberosum* L.) tubers caused by *Rhizoctonia solani*, wireworms (*Agriotes spp*.) and slugs (*Deroceras reticulatum, Arion hortensis*) in different farming systems. *Field Crops Research*, 128, 147-155. doi:10.1016/j.fcr.2012.01.004

Kim, J. R. et al., (2014). Diverse gastropod hosts of *Angiostrongylus cantonensis*, the rat lungworm, globally and with a focus on the Hawaiian Islands. *PLoS One,* 9(5), e94969. doi:10.1371/journal.pone.0094969

Koch, R., Jäckel, B., & Plate, H. P. (2000). Controlling pest slugs: New methods and the verification of their effects. *Gesunde Pflanzen*, 52, 1-10.

Kozłowski, J. (2012). The significance of alien and invasive slug species for plant communities in agrocenoses. *Journal of Plant Protection Research*, 52, 67-76.

Kralka, R.A., & Samuel, W.M. (1984). Experimental life cycle of *Protostrongylus boughtoni* (Nematoda: Metastrongyloidea), a lung worm of snowshoe hares, *Lepus americanus*. *Canadian Journal of Zoology*, 62, 473-479.

Lahmar, S., Cabaret, J., & Cheniti, T. (1990). Land snails and periods at high risk for protostrongylid infection on a sheep-grazed pasture of northeast Tunisia. *Veterinary Parasitology*, 36, 105-115.

Lange Jr, W. H., & Macleod, G. F. (1941). Metaldehyde and calcium arsenate in slug and snail baits. *Journal of Economic Entomology*, 34, 321-322.

Lima, W. D. S. et al., (2009). Occurrence of *Fasciola hepatica* (Linnaeus, 1758) infection in Brazilian cattle of Minas Gerais, Brazil. *Revista Brasileira de Parasitologia Veterinária*, 18, 27-30. doi:10.4322/rbpv.01802006

López, L. P., Romero, J., & Velásquez, L. E. (2008). Paramphistomidae isolation from dairy cows and its intermediate host (*Lymnaea truncatula* and *Lymnaea columella*) in a dairy farm in western Colombian high tropical region. *Revista Colombiana de Ciencias Pecuarias*, 21, 9-18.

Lovett, A.L., & Black, A.B. (1920). The gray garden slug: with notes on allied forms. *Station Bulletin*, 170, 1-43.

Maat‐Bleeker, F. et al., (1995). Vineyard snail allergy possibly induced by sensitization to house‐dust mite (*Dermatophagoides pteronyssinus*). *Allergy*, 50, 438-440.

Madsen, H., & Frandsen, F. (1989). The spread of freshwater snails including those of medical and veterinary importance. *Acta Tropica*. 46, 139-146.

Moran, S., Gotlib, Y., & Yaakov, B. (2004). Management of land snails in cut green ornamentals by copper hydroxide formulations. *Crop Protection*, 23, 647-650. doi:10.1016/j.cropro.2003.11.004

Morassutti, A. L. et al., (2014). Eosinophilic meningitis caused by *Angiostrongylus cantonensis*: An emergent disease in Brazil. *Memórias do Instituto Oswaldo Cruz*, 109, 399-407. doi:http://dx.doi.org/10.1590/0074-0276140023

Moreira, V. L. C. et al., (2013). Endemic angiostrongyliasis in the Brazilian Amazon: Natural parasitism of *Angiostrongylus cantonensis* in *Rattus rattus* and *R. norvegicus*, and sympatric giant African land snails, *Achatina fulica*. *Acta Tropica*, 125, 90-97. doi: https://doi.org/10.1016/j.actatropica.2012.10.001

Nash, M. A., & Hoffmann, A. A. (2012). Effective invertebrate pest management in dryland cropping in southern Australia: The challenge of marginality. *Crop Protection*, 42, 289-304. doi:http://dx.doi.org/10.1016/j.cropro.2012.06.017

Oliveira, A. P. et al., (2015). *Angiostrongylus cantonensis* infection in molluscs in the municipality of São Gonçalo, a metropolitan area of Rio de Janeiro, Brazil: Role of the invasive species *Achatina fulica* in parasite transmission dynamics. *Memórias do Instituto Oswaldo Cruz*, 110, 739-744. doi:http://dx.doi.org/10.1590/0074-02760150106

Oliveira, C. D., Vasconcellos, M. C., & Pinheiro, J. (2008). The population density effects on the reproductive biology of the snail *Bradybaena similaris* (Férussac, 1821)(Mollusca, Gastropoda). *Brazilian Journal of Biology*, 68, 367-371. doi:http://dx.doi.org/10.1590/S1519-69842008000200018

Pinder, L. C. V. (1974). The ecology of slugs in potato crops, with special reference to the differential susceptibility of potato cultivars to slug damage. *Journal of Applied Ecology*, 11, 439-451.

Pinto-Guillaume, E. M. (2002). Mollusks from the Villa of Livia at Prima Porta, Rome: The Swedish Garden Archaeological Project, 1996-1999. *American Journal of Archaeology*, 106, 37-58.

Pinto-Guillaume, E. M. (2008). Molluscs from the Villa of Livia revisited. *The Archaeo+ Malacology Group Newsletter*, 11, 1-13.

Pinto, H. A., Brant, S. V., & de Melo, A. L. (2014). *Physa marmorata* (Mollusca: Physidae) as a natural intermediate host of *Trichobilharzia* (Trematoda: Schistosomatidae), a potential causative agent of avian cercarial dermatitis in Brazil. *Acta Tropica*, 138, 38-43. doi:http://dx.doi.org/10.1016/j.actatropica.2014.06.002

Prystupa, B. D., Holliday, N. J., & Webster, G. R. B. (1987). Molluscicide efficacy against the marsh slug, *Deroceras laeve* (Stylommatophora: Limacidae), on strawberries in Manitoba. *Journal of Economic Entomology*, 80, 936-943.

Pullan, N. B., Climo, F. M., & Mansfield, C. B. (1972). Studies on the distribution and ecology of the family Lymnaeidae (Mollusca: Gastropoda) in New Zealand. *Journal of the Royal Society of New Zealand*, 2, 393-405.

Rambo, P. R., Agostini, A. A., & Graeff-Teixeira, C. (1997). Abdominal angiostrongylosis in southern Brazil - prevalence and parasitic burden in mollusc intermediate hosts from eighteen endemic foci. *Memórias do Instituto Oswaldo Cruz*, 92, 9-14.

Ree, R. V. et al., (1996). Asthma after consumption of snails in house‐dust‐mite‐allergic patients: A case of IgE cross‐reactivity. *Allergy*, 51, 387-393.

Rowley, M. A. et al., (1987). Terrestrial gastropod hosts of *Parelaphostrongylus tenuis* at the National Zoological Park's Conservation and Research Center, Virginia. *The Journal of Parasitology*, 73, 1084-1089.

Sallam, A., & El-Wakeil, N. (2012). Biological and ecological studies on land snails and their control. In S. Soloneski (Ed.), *Integrated pest management and pest control - current and future tactics* (pp. 413-444). [InTech]. Retrieved from http://www.intechopen.com/books/integrated-pest-managementand-pest-control-current-and-future-tactics/biological-and-ecological-studies-on-land-snails-and-their-control.

Salmijah, S. et al., (2006). Dose-response to metaldehyde and cytochrome P450 levels in *Bradybaena similaris* populations which have and have not had previous pesticide exposure. *Plant Protection Quarterly*, 21, 163-165.

Salvio, C. et al., (2008). The efficacy of three metaldehyde pellets marketed in Argentina, on the control of *Deroceras reticulatum* (Müller)(Pulmonata: Stylommatophora). *Spanish Journal of Agricultural Research*, 6, 70-77.

Schüder, I., Port, G., & Bennison, J. (2003). Barriers, repellents and antifeedants for slug and snail control. *Crop Protection*, 22, 1033-1038. doi:https://doi.org/10.1016/S0261-2194(03)00120-0

Senanayake, S. N. et al., (2003). First report of human angiostrongyliasis acquired in Sydney. *Medical Journal of Australia*, 179, 430-431.

Simms, L.C., Ester, A., & Wilson, M.J. (2006). Control of slug damage to oilseed rape and wheat with imidacloprid seed dressings in laboratory and field experiments. *Crop Protection*, 25, 549-555.

Spencer, H., & Stracener, C. L. (1929). Soil animals injurious to sugarcane roots. *Annals of the Entomological Society of America*, 22, 641-649.

Stockdale-Walden, H. D. et al., (2015). *Angiostrongylus cantonensis* in introduced gastropods in southern Florida. *Journal of Parasitology*, 101, 156-159. doi:https://doi.org/10.1645/14-553.1

Stojnić, B. et al., (2011). First record of introduced Valencia slug, *Lehmannia valentiana* (Férussac, 1822), in Serbia. *Pesticidi i Fitomedicina*, 26, 213-220. doi:10.2298/PIF1103213S

Swart, P. L., Barnes, B.N., & Myburgh, A.C. (1976). Pests of table grapes in the Western Cape. *Deciduous Fruit Grower*, 26, 169-195.

Symondson, W. O. C. (1989). Biological control of slugs by carabids. [Monograph]. *British Crop Protection Council,* 295-300.

Thiengo, S. C. (1995). RESEARCH NOTE Presence of *Strongyluris*-like Larvae (Nematoda) in some terrestrial molluscs in Brazil. *Memórias do Instituto Oswaldo Cruz*, 90, 619-620.

Thomas, A. K. (2010). *Impact of dietary diversification on invasive slugs and biological control with notes on slug species of Kentucky* (Unpublished master’s thesis). University of Kentucky, Kentucky, USA.

Thomas, D. C. (1947). Some observations on damage to potatoes by slugs. *Annals of Applied Biology*, 34, 246-251.

Toader-Williams, A., & Golubkina, N. (2009). Investigation upon the edible snail’s potential as source of selenium for human health and nutrition observing its food chemical contaminant risk factor with heavy metals. *Bulletin UASVM Agriculture*, 66, 495-499.

Ueno, H. et al., (1982). Fascioliasis problems in ruminants in Rio Grande do Sul, Brazil. *Veterinary Parasitology*, 11, 185-191.

Urban, E. (1980). Studies on lung nematodes (Protostrongylidae, Dictyocaulidae) in sheep of the Podhale region, Tatra highlands. II. intermediate hosts of Protostrongylidae. *Acta Parasitologica Polonica*, 27, 63-74.

Van Bruggen, A. C. (1964). The distribution of introduced mollusc species in Southern Africa. *Beaufortia*, 11, 161-169.

Walden, H. D. S. et al., (2017). Geographic distribution of *Angiostrongylus cantonensis* in wild rats (*Rattus rattus*) and terrestrial snails in Florida, USA. *PLoS One*, 12(5), e0177910. doi:https://doi.org/10.1371/journal.pone.0177910

Wiktor, A. (2001). *Deroceras (Deroceras) panormitanum* (Lessona et Pollonera, 1882) - a new introduced slug species in Poland (Gastropoda: Pulmonata: Agriolimacidae). *Folia Malacologica*, 9, 155-157.

Willis, J. C. et al., (2008). The importance of temperature and moisture to the egg‐laying behaviour of a pest slug, *Deroceras reticulatum*. *Annals of Applied Biology*, 153, 105-115. doi:10.1111/j.1744-7348.2008.00242.x

Wilson, M. J. et al., (1995). Biocontrol of slugs in protected lettuce using the rhabditid nematode *Phasmarhabditis hermaphrodita*. *Biocontrol Science and Technology*, 5, 233-242.

Wilson, M. J. et al., (1994). Biological control of slugs in winter wheat using the rhabditid nematode *Phasmarhabditis hermaphrodita*. *Annals of Applied Biology*, 125, 377-390.

Wilson, M. J. et al., (1996). Effects of soil incorporation on the efficacy of the rhabditid nematode, *Phasmarhabditis hermaphrodita*, as a biological control agent for slugs. *Annals of Applied Biology*, 128, 117-126.

Young, C. L. (2000). U.S. Patent No. 6,093,416. Washington, DC: U.S. Patent and Trademark Office.

Zhongzhang, T., & Chongti, T. (1997). The biology and epidemiology of *Eurytrema coelomaticum* (Giard et Billet, 1892) and *Eurytrema pancreaticum* (Janson, 1889) in cattle and sheep in China [J]. *Acta Zoologica Sinica*, 3, 004.
